# Identification and Prognostic Value Exploration of Radiotherapy Sensitivity-Associated Genes in Non-Small-Cell Lung Cancer

**DOI:** 10.1155/2021/5963868

**Published:** 2021-09-02

**Authors:** Qing Ma, Kai Geng, Ping Xiao, Lili Zeng

**Affiliations:** ^1^Department Oncology, Tianjin Medical University General Hospital, Tianjin 300052, China; ^2^Radiotherapy Department, Tianjin Medical University General Hospital, Tianjin 300052, China

## Abstract

**Background:**

Non-small-cell lung cancer (NSCLC) is a prevalent malignancy with high mortality and poor prognosis. The radiotherapy is one of the most common treatments of NSCLC, and the radiotherapy sensitivity of patients could affect the individual prognosis of NSCLC. However, the prognostic signatures related to radiotherapy response still remain limited. Here, we explored the radiosensitivity-associated genes and constructed the prognostically predictive model of NSCLC cases.

**Methods:**

The NSCLC samples with radiotherapy records were obtained from The Cancer Genome Atlas database, and the mRNA expression profiles of NSCLC patients from the GSE30219 and GSE31210 datasets were obtained from the Gene Expression Omnibus database. The Weighted Gene Coexpression Network Analysis (WGCNA), univariate, least absolute shrinkage and selection operator (LASSO), multivariate Cox regression analysis, and nomogram were conducted to identify and validate the radiotherapy sensitivity-related signature.

**Results:**

WGCNA revealed that 365 genes were significantly correlated with radiotherapy response. LASSO Cox regression analysis identified 8 genes, including FOLR3, SLC6A11, ALPP, IGFN1, KCNJ12, RPS4XP22, HIST1H2BH, and BLACAT1. The overall survival (OS) of the low-risk group was better than that of the high-risk group separated by the Risk Score based on these 8 genes for the NSCLC patients. Furthermore, the immune infiltration analysis showed that monocytes and activated memory CD4 T cells had different relative proportions in the low-risk group compared with the high-risk group. The Risk Score was correlated with immune checkpoints, including CTLA4, PDL1, LAG3, and TIGIT.

**Conclusion:**

We identified 365 genes potentially correlated with the radiotherapy response of NSCLC patients. The Risk Score model based on the identified 8 genes can predict the prognosis of NSCLC patients.

## 1. Introduction

Lung cancer serves as the most prevalent malignancy and is the leading cause of tumor-associated mortality globally, according to the latest annual statistics report of global cancer [1]. Non-small-cell lung cancer (NSCLC) accounts for about 83% of primary lung cancer [2]. NSCLC is a prevalent cancer type with high incidence and severe mortality [3]. Although great advancements in surgical and therapeutic interventions have been achieved, the prognosis of NSCLC cases remains poor, and the recurrence rate of the subjects is high due to the treatment resistance or tumor metastasis [4]. Therefore, the exploration of prognostic biomarkers, especially those that are closely correlated with the treatment response, will benefit the selection and development of therapeutic strategy for NSCLC.

The therapeutic strategy of NSCLC involves various treatment modalities, including surgery, systemic therapies, and radiotherapy [5]. Radiotherapy is an essential modality of NSCLC treatment, and 77% of NSCLC patients have an evidence-based indication for radiotherapy, although it is often under resistance or underutilized [6]. Consequently, the radiotherapy response significantly affects the prognosis and thereby determines the therapy decision of NSCLC patients [7, 8]. The exploration of radiotherapy response-related genetic factors is helpful for the NSCLC treatment selection and development of the combinational therapeutic strategy [9, 10]. Several researches have explored the radiotherapy response of NSCLC through gene expression profiling. Chen et al. found that PAF was upregulated in NSCLC samples in comparison to the normal controls, which was associated with the poor prognosis of NSCLC patients, and the radiosensitivity of NSCLC cells could be improved with the decrease of PAF expression level [11]. Four microRNAs, including hsa-miR-98-5p, hsa-miR-495-3p, hsa-miR-302e, and hsa-miR-613, were identified as potential biomarkers for NSCLC radiosensitivity based on the gene expression profiles and tumor response criterion [12]. The elevated expression level of miR-449a could enhance the DNA damage and apoptosis induced by irradiation in NSCLC cell lines and contributed to the radiosensitivity of NSCLC [13]. The glutamine metabolism genes, including ME1 and GOT1, were identified as novel indicators for NSCLC radioresistance and predictive biomarkers in the radiation treatment of NSCLC [14]. However, to our knowledge, few studies have explored the prognostic value of radiotherapy response-related genes in NSCLC *via* the integration of multiple genetic factors and establishment of predictive model by machine learning.

Combining several biomarkers into a single model will substantially enhance the prognostic value [15–17]. The discovery of prognostic biomarkers and signatures requires multiple practical bioinformatic methods. The least absolute shrinkage and selection operator method (LASSO) is a standard method for the regression of high-dimensional predictors [18, 19]. LASSO has been broadly applied to the Cox proportionate hazard regression model for the prognostic analysis of high-dimensional data [20]. Besides, the Weighted Gene Coexpression Network Analysis (WGCNA) is a helpful tool to investigate the relationship between gene expression and clinical traits in many malignancies including NSCLC [21]. In addition, the nomogram is a prevalently utilized tool in oncology, which is able to build a particular probability by integrating different determinant and prognostic variables according to corresponding clinical features [22]. However, the application of these bioinformatic tools in the exploration of prognostic signature related to radiotherapy response of NSCLC patients is still limited.

In this study, we were interested in integrating the analysis of mRNA expression profiling data of NSCLC samples that have undergone radiotherapy and bioinformatics to identify the prognostic value of radiotherapy sensitivity-associated genes in NSCLC. A total of 365 genes potentially correlated with the radiotherapy response of NSCLC patients were identified, and 8 genes were selected for the Risk Score model. The Risk Score model as well as the nomogram model based on the Risk Score and smoking status could reliably predict the prognosis of NSCLC patients.

## 2. Materials and Methods

### 2.1. Data Collection

The mRNA expression profiling data of 493 lung squamous cell carcinoma (LUSC) samples and 500 lung adenocarcinoma (LUAD) samples with complete survival information were obtained from The Cancer Genome Atlas database (TCGA, https://tcga-data.nci.nih.gov/tcga/). The detailed clinical information of the samples is shown in Table 1.

The mRNA expression profiling data of the GSE30219 dataset contained 307 NSCLC patients, in which 274 patients had complete survival information. The mRNA expression profiling data of the GSE31210 dataset contained 246 NSCLC patients, in which 226 patients had complete survival information. The expression of genes was analyzed using the Affymetrix Human Genome U133 Plus 2.0 Array platform. The samples with incomplete survival information were excluded, while samples with complete survival information were retained for further analysis.

### 2.2. WGCNA

WGCNA was performed by the *WGCNA R* package in the NSCLC samples with a clear record of radiotherapy response from TCGA database [23]. The hierarchical cluster was conducted based on the gene expression of the sample. The modules were identified using the dynamic cut tree method, and the genes with high similarity were classified into the same module. The module eigengene (ME) value of each module and the correlation coefficient of the ME value with the phenotype, including age (continuous phenotype), gender (binary classification phenotype), and the patient's response to radiotherapy (continuous phenotype), were calculated. The minimum number of genes was set as 50 for each module, and the height was set as 0.25.

### 2.3. Gene Ontology (GO) and Kyoto Encyclopedia of Genes and Genomes (KEGG) Analysis

GO and KEGG pathway analyses were performed by using *clusterProfiler* package of *R* [24]. The GO included molecular function (MF), biological process (BP), and cellular component. *P* < 0.05 was regarded as statistically significant.

### 2.4. LASSO Cox Regression Analysis

The correlation of gene expression with overall survival (OS) of NSCLC patients was evaluated by univariate Cox regression, in which the threshold was *P* < 0.05. The LASSO Cox regression analysis was performed to further optimize the genes associated with the prognosis of NSCLC using the *glmnet R* package [25]. The Risk Score was calculated based on the selected genes using the following formula:

(1) $$\begin{array}{c} \text{Risk Score}=\overset{n}{\underset{i=1}{\sum }}{\text{Coef}}_{i}*x_{i}. \end{array}$$

Coef_*i*_ was the risk coefficient of each factor calculated by the LASSO Cox model, and *X*_*i*_ was the mRNA expression value of the selected genes. Herein, we standardized the expression values of the selected genes to the data with the average value of 0 and standard deviation of 1. The optimal cutoff value of Risk Score was identified by *survival R* package, *survminer R* package, and two-sided log-rank test [26]. Then, the samples were separated into a low-risk group and high-risk group based on the cutoff value.

### 2.5. Survival Analysis

The OS was analyzed by the *survival* and *survminer R* packages based on the Kaplan-Meier method [26], and the difference in the OS of distinct groups was evaluated by the two-sided log-rank test. To further evaluate whether the Risk Score was an independent signature, we performed the multivariate Cox regression analysis, containing age, TNM stage, gender, smoking status, and the Risk Score for the LUSC samples that have undergone radiotherapy from TCGA.

### 2.6. Analysis of Immune Infiltration

The immune infiltration of 22 immune cells in the samples was analyzed by using CIBERSORT software combined with the LM22 feature matrix [27]. The sum of the proportions of all estimated immune cell types in each sample is equal to 1.

### 2.7. Nomogram Construction

A concise nomogram for survival prediction of the NSCLC patients was established using the *rms R* package based on the independent factors identified by multivariate Cox regression analysis [28]. The calibration curve of the nomogram was obtained, and the relationship between the predicted probability of nomogram and the actual incidence rate was observed. *P* < 0.05 was considered statistically significant.

### 2.8. Statistical Analysis

The OS derived from Kaplan-Meier-based survival analysis of distinct groups was compared by the two-sided log-rank test. The difference in the immune cell infiltration of the samples was analyzed by the Wilcoxon rank-sum test with the threshold of *P* < 0.05. The statistical analysis was performed by *R* software (version v3.5.2).

## 3. Results

### 3.1. WGCNA and Functional Enrichment Analysis

**Figure 1:**
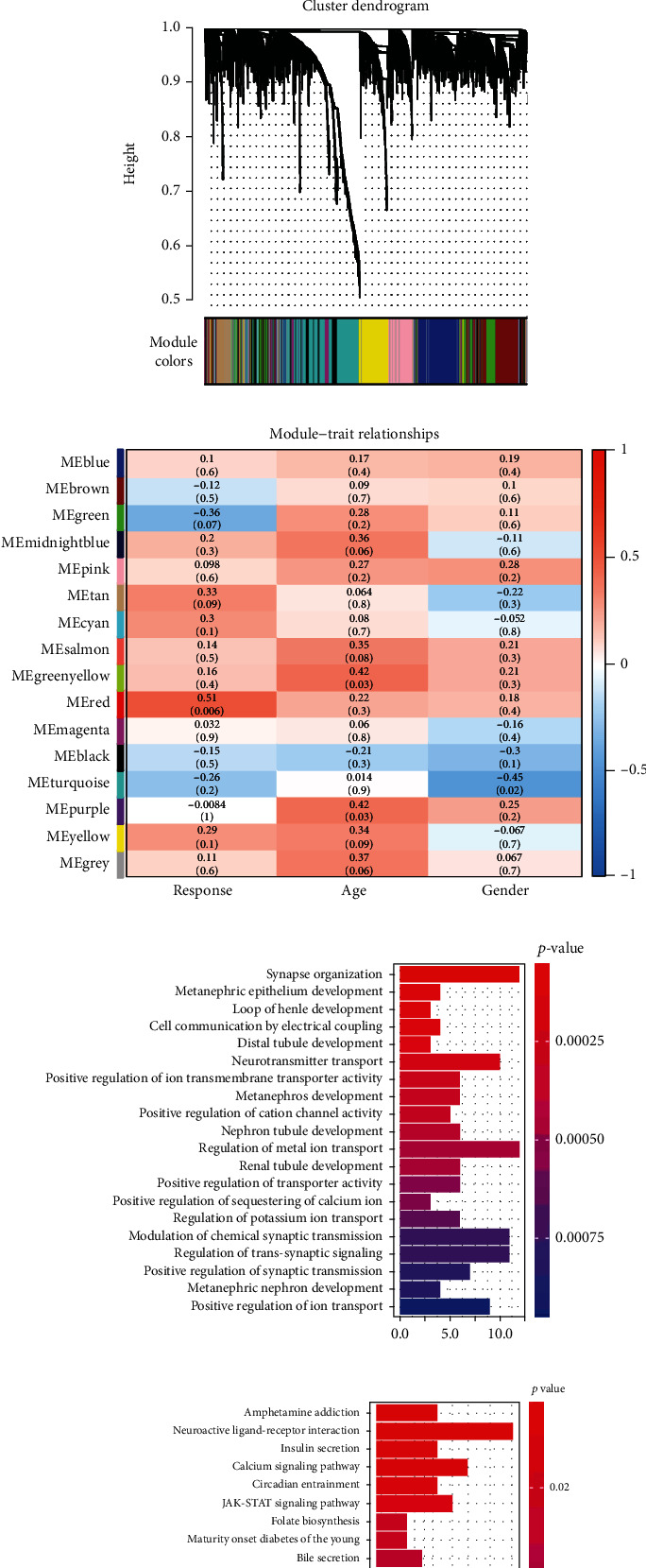
WGCNA and functional enrichment analysis. (a) The cluster analysis was performed in 27 NSCLC samples with clear record of radiotherapy response from TCGA database. (b) Selection of the soft threshold. The red line was the correlation coefficient, and the power value of *β* = 5 was selected as the soft threshold to construct a scale-free network. The left panel showed the scale-free fit index (*y*-axis) as a function of the soft threshold power (*x*-axis). The right panel displayed the mean connectivity (degree, *y*-axis) as a function of the soft threshold power (*x*-axis). (c) The clustering dendrograms of genes were shown, with assigned module colors. The gray module contained the genes that could not be clustered into other modules. (d) The correlation of the modules with the clinical traits, including age, stage, and radiotherapy, was shown in the heat map, with corresponding correlation coefficient and *P* value. (e) The top 20 significantly enriched GO terms. The *y*-axis was the GO terms, and the *x*-axis was the gene numbers. (f) The top 20 significant enriched KEGG pathways. The *y*-axis was the KEGG pathways, and the *x*-axis was the gene number.

To evaluate the correlation of potential genes with radiotherapy response of NSCLC patients, 27 NSCLC samples with clear record of radiotherapy response from TCGA database (Table S1) were used to establish the coexpression module by WGCNA. The cluster analysis showed that there were no outliers in the samples (Figure 1(a)). Besides, the power value of *β* = 5 (scale-free *R*^2^ = 0.90) was selected as the soft threshold to construct a scale-free network (Figure 1(b)). A total of 16 modules were identified through the average linkage hierarchical clustering (Figure 1(c)). The correlation of the modules with clinical traits, including age, stage, and radiotherapy, was analyzed. The red module, which contained 365 genes (Table S2), showed the highest correlation with radiotherapy (correlation coefficient = 0.51, *P* = 0.006) (Figure 1(d)), and this module was selected for further analysis.

Then, the GO and KEGG pathway analyses were performed. Multiple GO terms, including synapse organization, and KEGG pathways, including amphetamine addiction, were markedly enriched based on the 365 genes (Table S3 and S4), and the top 20 remarkable GO terms and KEGG pathways are demonstrated in Figures 1(e) and 1(f). The results implied that these cellular processes might be related to the radiotherapy response of NSCLC patients.

### 3.2. Construction and Validation of Prognosis Model

**Figure 2:**
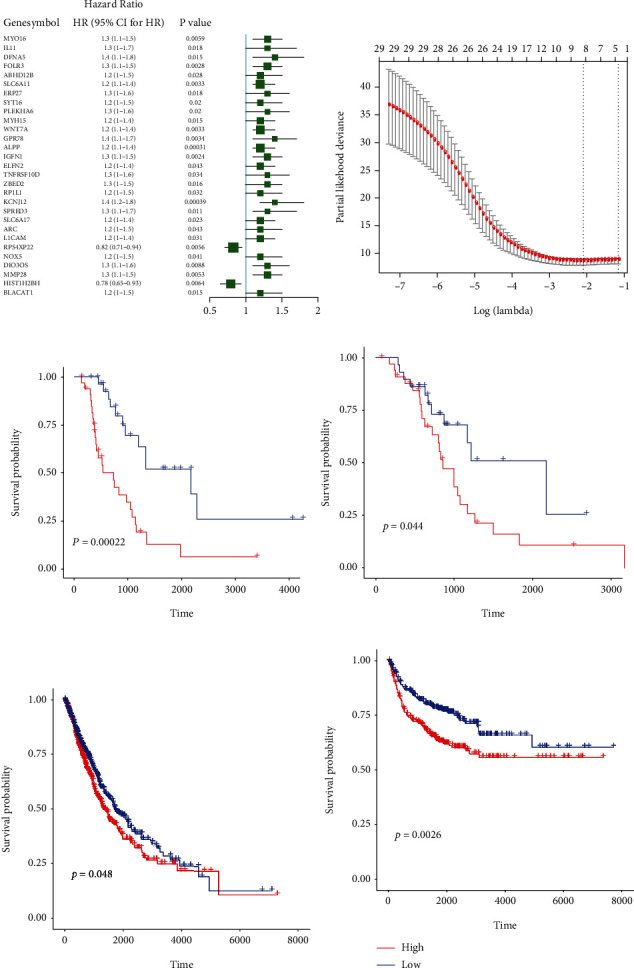
Construction and validation of the prognosis model for NSCLC. (a) A total of 29 genes were identified by univariate Cox regression analysis in 65 samples who have undergone radiotherapy from the LUSC dataset of TCGA. HR represented hazard ratio, and 95% CI represented 95% confidence interval. (b) The optimal lambda value was selected in the LASSO Cox regression analysis. (c, d) The LUSC samples and LUAD samples that have undergone radiotherapy from TCGA were separated into high-risk and low-risk groups based on the best cutoff point (0.2153), respectively. (c) The OS analysis was performed in the LUSC dataset. (d) The OS analysis was conducted in the LUAD dataset. The *P* value was assessed by the two-sided log-rank test. (e, f) All the samples with or without radiotherapy from TCGA cohort including LUSC and LUAD samples and GEO cohort including GSE30219 and GSE31210 were separated into high-risk and low-risk groups. (e) The OS analysis was conducted in the TCGA cohort. (f) The OS analysis was performed in the GEO cohort. The *P* value was assessed by the two-sided log-rank test.

Given that the radiotherapy response of patients is closely associated with prognosis, we further constructed the prognostic model based on the identified genes, in which the LUSC samples from TCGA served as the training dataset, and the LUAD samples from TCGA, GSE30219, and GSE31210 cohorts from GEO database served as the validation datasets. A total of 65 samples that have undergone radiotherapy from the TCGA-LUSC dataset were subjected to univariate Cox regression analysis, in which the expression of the identified 365 genes served as the continuous variable. Our data revealed that 29 genes were significantly associated with the OS of the samples (*P* < 0.05) (Figure 2(a)). These significant genes were selected for LASSO Cox regression analysis, and the model achieved the best performance with 8 genes, including FOLR3, SLC6A11, ALPP, IGFN1, KCNJ12, RPS4XP22, HIST1H2BH, and BLACAT1 (Figure 2(b)). The expressions of these 8 genes in samples with different radiotherapy response status were analyzed (Figure S1). The Risk Score model was then established: Risk Score = 0.015771657∗expression value of FOLR3 + 0.060997544∗expression value of SLC6A11 + 0.026587925∗expression value of ALPP + 0.036075351∗expression value of IGFN1 + 0.156648675∗expression value of KCNJ12 − 0.002265927∗expression value of RPS4XP22 − 0.116358137∗expression value of HIST1H2BH + 0.011770327∗expression value of BLACAT1. The LUSC and LUAD samples that have undergone radiotherapy from TCGA were separated to high-risk and low-risk groups based on the best cutoff point (0.2153), respectively. The survival analysis revealed that the OS of the low-risk group was better than that of the high-risk group (Figures 2(c) and 2(d)). Next, all samples with or without radiotherapy from TCGA cohort including the LUSC and LUAD samples and GEO cohort including GSE30219 and GSE31210 datasets were separated into the high-risk and low-risk groups, respectively. The survival analysis demonstrated that the OS of the low-risk group was better than that of the high-risk group (Figures 2(e) and 2(f)). Together, these data suggested that the Risk Score model based on the identified 8 genes could reliably predict the prognosis of NSCLC patients with or without radiotherapy.

### 3.3. Multivariate Cox Regression Analysis of the Signature

**Figure 3:**
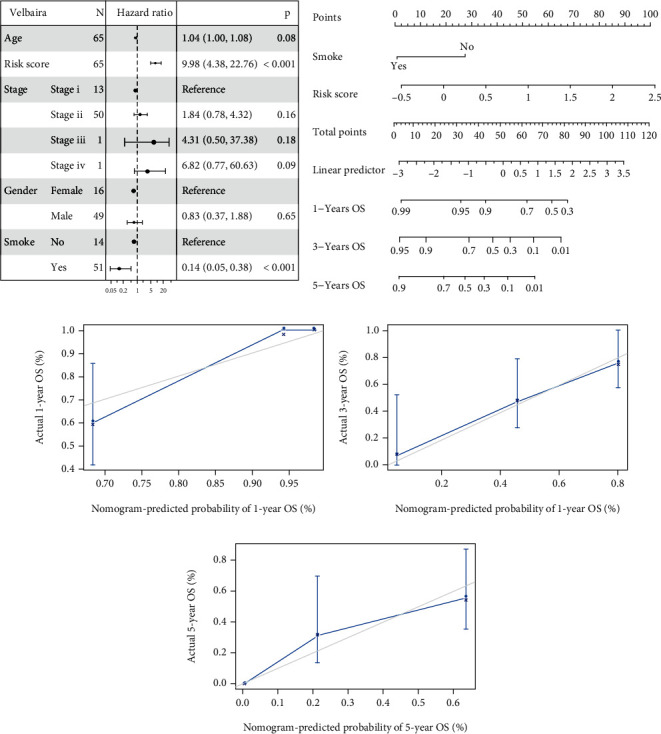
Multivariate Cox regression analysis of the signature and nomogram construction. (a) The multivariate Cox regression analysis, containing age, TNM stage, gender, smoking status, and the Risk Score, was performed in the LUSC samples that have undergone radiotherapy from TCGA. Compared with the reference samples, the samples with hazard ratio larger than 1 represented a higher risk of death, and the samples with hazard ratio less than 1 represented a lower risk of death. (b) The nomogram model based on Risk Score and smoking status was constructed to predict 1-year, 3-year, and 5-year OS of NSCLC patients. (c–e) The calibration curves of the nomogram for the estimation of survival rates at 1 year, 3 years, and 5 years were shown, respectively. The *x*-axis represented the predicted survival rate of the nomogram, and the *y*-axis represented the actual survival rate.

To further evaluate whether the Risk Score was an independent signature, we performed the multivariate Cox regression analysis for the LUSC samples that have undergone radiotherapy from TCGA, and multiple factors containing age, TNM stage, gender, smoking status, and the Risk Score were incorporated. Our data confirmed that the Risk Score and smoking were significant independent risk factors in the system (HR = 9.98; 95% CI [4.38-22.76]; *P* < 0.001) (Figure 3(a)), suggesting that Risk Score could independently predict the prognosis of NSCLC patients who have undergone radiotherapy.

### 3.4. Nomogram Construction

Next, we constructed a nomogram model based on the independent prognostic signatures, including the Risk Score and smoking status (Figure 3(b)). The results showed that the nomogram presented good performance in predicting the 1-year, 3-year, and 5-year OS of NSCLC patients who have undergone radiotherapy (Figures 3(c)–3(e)).

### 3.5. Immune Landscape between the Low- and High-Risk NSCLC Patients

**Figure 4:**
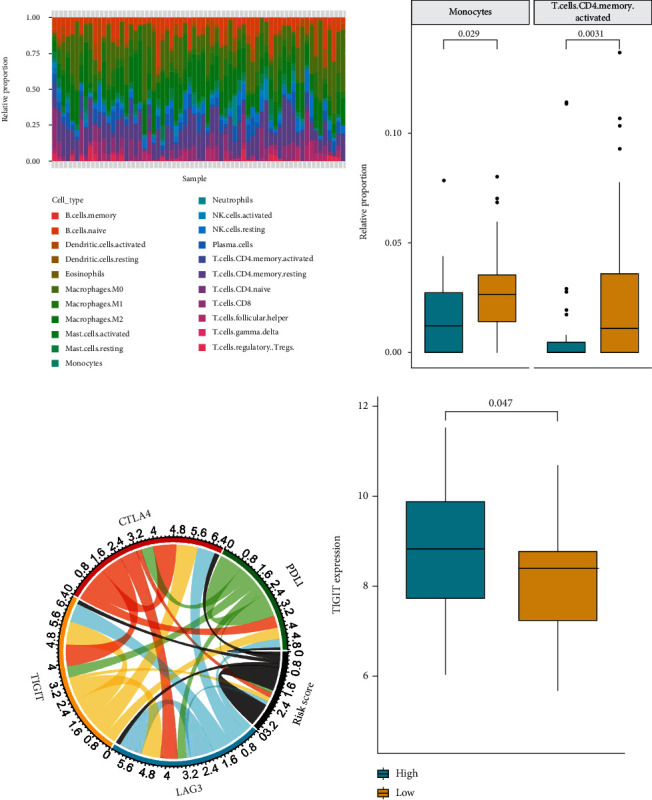
Immune landscape between the low- and high-risk NSCLC patients. (a) The immune infiltration difference of 22 immune cells in 65 LUSC patients who have undergone radiotherapy from TCGA. (b) The infiltration difference of monocytes and activated memory CD4 T cells between the low-risk group and the high-risk group was analyzed. The difference of immune cell infiltration in the samples was analyzed by the Wilcoxon rank-sum test with the threshold of *P* < 0.05. (c) The correlation of the Risk Score with the expression of the 4 key immune checkpoints, including CTLA4, PDL1, LAG3, and TIGIT. (d) The expression of TIGIT in the low-risk group and the high-risk group of NSCLC patients with radiotherapy.

Then, the immune infiltration of 22 immune cells in 65 LUSC patients who have undergone radiotherapy from TCGA was analyzed using CIBERSORT software combined with the LM22 feature matrix (Figure 4(a)). The significant difference of monocytes and activated memory CD4 T cells was identified in the low-risk group compared with the high-risk group (Figure 4(b)).

The expression of immune checkpoints has become a biomarker for the immunotherapy selection of NSCLC patients [29–31]. Therefore, we analyzed the correlation between the Risk Score and the key immune checkpoints, including CTLA4, PDL1, LAG3, and TIGIT. Our data showed that the Risk Score was significantly correlated with these immune checkpoints (Figure 4(c)). Meanwhile, the expression of TIGIT in the high-risk group of NSCLC patients with radiotherapy was significantly higher than that in the low-risk group (*P* < 0.05) (Figure 4(d)), implying that immunosuppressive microenvironment might contribute to the poor prognosis of NSCLC patients with radiotherapy.

## 4. Discussion

NSCLC is the predominant type of lung cancer with high mortality and poor prognosis [32]. Radiotherapy plays a key role in both curative and palliative treatments of NSCLC [33]. Increasing evidence has revealed that the genetic factors are closely associated with the radiotherapy response of NSCLC patients. For example, it has been reported that the abnormal expression of RAD50 is correlated with the poor clinical outcome during radiotherapy for NSCLC [34]. Potentially functional ATG16L2 variants foretell radiation outcomes and pneumonitis in NSCLC patients after radiotherapy [35]. The expression of UNC5A is associated with the prognosis of radiotherapy and clinicopathologic features in NSCLC patients [36]. However, few studies have explored the prognostic value of radiotherapy response-related genes in NSCLC through integrating multiple significant genes and establishing predictive models *via* machine learning.

In this study, we performed WGCNA for the NSCLC samples with a clear record of radiotherapy response from TCGA database and identified 16 modules. The red module, which contained 365 genes, was observed to have the highest correlation with radiotherapy response. A univariate Cox regression analysis was performed on the 65 samples that have undergone radiotherapy from TCGA-LUSC dataset, and the result revealed that 29 genes were significantly associated with the OS of the samples. LASSO Cox regression analysis demonstrated that 8 genes, including FOLR3, SLC6A11, ALPP, IGFN1, KCNJ12, RPS4XP22, HIST1H2BH, and BLACAT1, were closely associated with the prognosis of NSCLC patients. Among the 8 genes, FOLR3 is reported to correlate with an enhanced likelihood of NSCLC progression at midtreatment radiological evaluation [37]. BLACAT1 contributes to the modulation of progression, epithelial-mesenchymal transition, and metastasis of NSCLC by targeting Wnt/*β*-catenin signaling [38]. SLC6A11 is a member of the solute carrier family 6 [39]. Although there is no direct research on the role of SLC6A11 in NSCLC, another member of this family, SLC6A10P, is found to be overexpressed in NSCLC tissues compared with the normal lung tissues, serving as a predictor of poor prognosis in NSCLC [40]. In addition, SLC6A15 shows high mRNA expression and protein levels in NSCLC cell lines, being considered a potential metabolic target for NSCLC [41]. There have not been studies in regard to the effects of the remaining 5 genes on NSCLC, but most of them are proved to be associated with other cancers. ALPP encodes an alkaline phosphatase, and its increased expression has been observed in several cancers, including breast cancer and intratubular germ cell neoplasia [42, 43]. Frequent IGFN1 mutation is detected in metastatic breast cancer, the disease which leads to the poor prognosis of patients [44]. KCNJ12 shows high frequency of single-nucleotide polymorphisms in head and neck squamous cell carcinomas [45] and is adopted for stratifying the stage of colorectal cancer [46]. HIST1H2BH is related to the survival outcome of cervical cancer patients, which could be used for prognosis prediction [47]. However, the association of these genes with NSCLC still needs further study.

Importantly, the OS of the low-risk group was better than that of the high-risk group separated by the Risk Score based on these 8 genes in the NSCLC patients with or without radiotherapy. The multivariate Cox regression analysis further confirmed that the Risk Score, along with the smoking status, was an independent predictive signature in the system. The nomogram model based on the Risk Score showed good performance in predicting the survival of NSCLC patients. These data suggest that the Risk Score model based on the identified 8 genes can reliably predict the prognosis of NSCLC patients with or without radiotherapy. Several prognostic models for NSCLC have been previously established. Ganti et al. recruited 1467 patients (≥70 years old) with advanced NSCLC and incorporated the factors including gender, inferior performance status, weight loss, and metastasis status into the predictive model for the prognosis of older patients with advanced NSCLC [48]. Park et al. constructed an iSEND model, which consisted of immunotherapy, sex, Eastern Cooperative Oncology Group performance status, neutrophil-to-lymphocyte ratio (NLR), and delta NLR, to predict the prognosis of patients with NSCLC who have been treated with nivolumab [49]. Laar investigated the genetic signatures to predict the prognosis and chemotherapy benefit of NSCLC patients through the application of 160-gene and 37-gene signatures, respectively [50]. Compared with these three models, our research established the predictive model for NSCLC prognosis using a signature with less genes and could be more applicable for NSCLC, which was not affected by the stage and treatment of NSCLC patients.

The combination of radiotherapy and immunotherapy improves the treatment effect on NSCLC, due to the fact that the immune microenvironment plays a crucial role in the development of NSCLC and the radiotherapy response of NSCLC patients [51, 52]. It has been reported that the lymphocyte-monocyte ratio serves as a biomarker to predict the prognosis of NSCLC with radiotherapy [53]. The memory CD4+ T cells in NSCLC patients are the predictor of radiotherapy response [54]. Moreover, immune checkpoints participate in the modulation of NSCLC progression and the expression of immune checkpoints is associated with the radiotherapy sensitivity of NSCLC patients. Our immune infiltration analysis revealed increased relative proportions of infiltrating monocytes and activated memory CD4 T cells in the high-risk group with inferior prognosis compared with the low-risk group with superior prognosis separated by the Risk Score based on these 8 genes in the NSCLC patients who have undergone radiotherapy. It has been known that the monocytes could produce tissue factor in NSCLC, which enhances the proliferative and metastatic capacities of tumor cells, increases the degree of malignancy, and is related to the reduced survival rate of NSCLC patients [55, 56]. Thus, it is speculated that the increased monocyte proportion may contribute to poor prognosis of NSCLC by generating tumor factor. Moreover, the elevated memory CD4 T cells in the high-risk group with inferior prognosis is consistent with the previous study of Liu et al., which demonstrates that the increased memory CD4 T cells are correlated with the poor prognosis of NSCLC patients after radiotherapy [57]. However, the underlying mechanism between monocytes, activated memory CD4 T cells, and the prognosis of NSCLC patients still needs further investigation. The Risk Score was remarkably correlated with the immune checkpoints, including CTLA4, PDL1, LAG3, and TIGIT. The expression of TIGIT in the high-risk group of NSCLC patients with radiotherapy was significantly higher than that in the low-risk group. These data suggest that the immunosuppressive microenvironment may contribute to the poor prognosis of NSCLC patients after radiotherapy.

In conclusion, this study identified 365 genes potentially correlated with the radiotherapy response of NSCLC patients. The Risk Score model based on the identified 8 genes, including FOLR3, SLC6A11, ALPP, IGFN1, KCNJ12, RPS4XP22, HIST1H2BH, and BLACAT1, can reliably predict the prognosis of NSCLC patients with or without radiotherapy. Our finding provides a valuable prognostic model, benefiting the prognosis prediction of NSCLC patients.

However, there are some limitations in our study: (1) the 365 genes related to radiotherapy response in the red module could not be further distinguished as radiosensitive genes and radioresistant genes based on current information; (2) due to the lack of samples, the performance of the prognostically predictive model has not been validated with the clinical samples from our institute; and (3) in contrast to the other predictive models for the prognosis of NSCLC patients, only 27 NSCLC samples have clear record of radiotherapy response and are used to screen the radiotherapy response-related module, from which the 8 genes of the prognostic signature are derived. Further studies with larger sample size and more detailed radiotherapy response information are necessarily required. We will also be devoted to collecting more clinical samples from our institute for the validation of our results.

## Figures and Tables

**Table 1:** Clinicopathological characteristics of NSCLC patients from TCGA database.

Characteristics		LUAD patients (*N* = 500)		LUSC patients (*N* = 493)	
		No.	%	No.	%
Age	≤median	247	49.40%	276	55.98%
	>median	253	50.60%	217	44.02%
Smoke	Yes	342	68.40%	418	84.79%
	No	158	31.60%	75	15.21%
Sex	Female	270	54.00%	128	25.96%
	Male	230	46.00%	365	74.04%
Race	White	286	57.20%	348	70.59%
	Black or African American	52	10.40%	29	5.88%
	Asian	7	1.40%	9	1.83%
	American Indian or Alaska Native	1	0.20%	0	0.00%
	Unknown	54	10.80%	107	21.70%
Pathologic stage	i	268	53.60%	241	48.88%
	ii	119	23.80%	158	32.05%
	iii	80	16.00%	83	16.84%
	iv	25	5.00%	7	1.42%
	Unknown	8	1.60%	4	0.81%
Survival time	Long (>5 years)	52	10.40%	84	17.04%
	Short (<5 years)	448	89.60%	409	82.96%
Radiotherapy	Yes	62	12.40%	65	13.18%
	No	65	13.00%	77	15.62%
	Unknown	373	74.60%	351	71.20%
Chemotherapy	Yes	136	27.20%	109	22.11%
	No	78	15.60%	79	16.02%
	Unknown	286	57.20%	305	61.87%
OS status	Dead	182	36.40%	211	42.80%
	Alive	318	63.60%	282	57.20%

## Data Availability

In this study, our mRNA expression profiling data were downloaded from the GSE30219 dataset, the GSE31210 dataset, and The Cancer Genome Atlas database (TCGA, https://tcga-data.nci.nih.gov/tcga/).

## Additional Points

*Code Availability*. We mainly use two softwares: R language and CIBERSORT. There are mainly the *WGCNA* R package, *cluster Profiler* package, the *glmnet* R package, *survival* R package, *survminer* R package, and the *rms R* package in R language.

## Supplementary Materials

Supplementary 1 Figure S1: the expressions of FOLR3, SLC6A11, ALPP, IGFN1, KCNJ12, RPS4XP22, HIST1H2BH, and BLACAT1 in NSCLC samples with different radiotherapy response status.

Supplementary 2 Table S1: clinical information of samples with radiotherapy response information. Table S2: genes in the red module. Table S3: the information of GO analysis based on the identified genes. Table S4: the information of KEGG analysis based on the identified genes.

## References

[1] Bray F., Ferlay J., Soerjomataram I., Siegel R. L., Torre L. A., Jemal A. (2018). Global cancer statistics 2018: GLOBOCAN estimates of incidence and mortality worldwide for 36 cancers in 185 countries. *CA: a Cancer Journal for Clinicians*.

[2] Hua Q., Mi B., Xu F. (2020). Hypoxia-induced lncRNA-AC020978 promotes proliferation and glycolytic metabolism of non-small cell lung cancer by regulating PKM2/HIF-1*α* axis. *Theranostics*.

[3] Voelker R. (2020). Targeted therapy and diagnostic test for non-small cell lung cancer. *Journal of the American Medical Association*.

[4] Kim C., Giaccone G. (2018). Precision oncology in non-small-cell lung cancer: opportunities and challenges. *Nature Reviews. Clinical Oncology*.

[5] Vinod S. K., Hau E. (2020). Radiotherapy treatment for lung cancer: current status and future directions. *Respirology*.

[6] Vinod S. K. (2015). International patterns of radiotherapy practice for non-small cell lung cancer. *Seminars in Radiation Oncology*.

[7] Kalman N. S., Weiss E., Walker P. R., Rosenman J. G. (2018). Local radiotherapy intensification for locally advanced non-small-cell lung cancer - a call to arms. *Clinical Lung Cancer*.

[8] Brada M., Ball C., Mitchell S., Forbes H., Ashley S. (2019). Improving outcomes in non-small cell lung cancer; population analysis of radical radiotherapy. *Radiotherapy and Oncology*.

[9] Ye L., Shi S., Zeng Z., Huang Y., Hu Y., He J. (2018). Nomograms for predicting disease progression in patients of stage I non-small cell lung cancer treated with stereotactic body radiotherapy. *Japanese Journal of Clinical Oncology*.

[10] Christodoulou M., Bayman N., McCloskey P., Rowbottom C., Faivre-Finn C. (2014). New radiotherapy approaches in locally advanced non-small cell lung cancer. *European Journal of Cancer*.

[11] Chen Y., Jin Y., Ying H., Zhang P., Chen M., Hu X. (2021). Synergistic effect of PAF inhibition and X-ray irradiation in non-small cell lung cancer cells. *Strahlentherapie und Onkologie*.

[12] Chen X., Xu Y., Liao X. (2016). Plasma miRNAs in predicting radiosensitivity in non-small cell lung cancer. *Tumour Biology*.

[13] Liu Y. J., Lin Y. F., Chen Y. F. (2013). MicroRNA-449a enhances radiosensitivity in CL1-0 lung adenocarcinoma cells. *PLoS One*.

[14] Chakrabarti G. (2015). Mutant *KRAS* associated malic enzyme 1 expression is a predictive marker for radiation therapy response in non-small cell lung cancer. *Radiation Oncology*.

[15] Ågesen T. H., Sveen A., Merok M. A. (2012). ColoGuideEx: a robust gene classifier specific for stage II colorectal cancer prognosis. *Gut*.

[16] Zhang J. X., Song W., Chen Z. H. (2013). Prognostic and predictive value of a microRNA signature in stage II colon cancer: a microRNA expression analysis. *The Lancet Oncology*.

[17] Halabi S., Lin C. Y., Small E. J. (2013). Prognostic model predicting metastatic castration-resistant prostate cancer survival in men treated with second-line chemotherapy. *Journal of the National Cancer Institute*.

[18] Daghir-Wojtkowiak E., Wiczling P., Bocian S. (2015). Least absolute shrinkage and selection operator and dimensionality reduction techniques in quantitative structure retention relationship modeling of retention in hydrophilic interaction liquid chromatography. *Journal of Chromatography. A*.

[19] Jiang Y., Zhang Q., Hu Y. (2018). ImmunoScore signature: a prognostic and predictive tool in gastric cancer. *Annals of Surgery*.

[20] Mavaddat N., Michailidou K., Dennis J. (2019). Polygenic risk scores for prediction of breast cancer and breast cancer subtypes. *American Journal of Human Genetics*.

[21] Niemira M., Collin F., Szalkowska A. (2019). Molecular signature of subtypes of non-small-cell lung cancer by large-scale transcriptional profiling: identification of key modules and genes by weighted gene co-expression network analysis (WGCNA). *Cancers (Basel)*.

[22] Wang W., Zhuang R., Ma H. (2020). The diagnostic value of a seven-autoantibody panel and a nomogram with a scoring table for predicting the risk of non-small-cell lung cancer. *Cancer Science*.

[23] Wan Q., Tang J., Han Y., Wang D. (2018). Co-expression modules construction by WGCNA and identify potential prognostic markers of uveal melanoma. *Experimental Eye Research*.

[24] Yu G., Wang L. G., Han Y., He Q. Y. (2012). clusterProfiler: an R package for comparing biological themes among gene clusters. *OMICS*.

[25] Friedman J., Hastie T., Tibshirani R. (2010). Regularization paths for generalized linear models via coordinate descent. *Journal of Statistical Software*.

[26] Li M., Spakowicz D., Burkart J. (2019). Change in neutrophil to lymphocyte ratio during immunotherapy treatment is a non-linear predictor of patient outcomes in advanced cancers. *Journal of Cancer Research and Clinical Oncology*.

[27] Newman A. M., Liu C. L., Green M. R. (2015). Robust enumeration of cell subsets from tissue expression profiles. *Nature Methods*.

[28] Li W., Liu J., Zhao H. (2020). Identification of a nomogram based on long non-coding RNA to improve prognosis prediction of esophageal squamous cell carcinoma. *Aging (Albany NY)*.

[29] Vansteenkiste J., Wauters E., Reymen B., Ackermann C. J., Peters S., De Ruysscher D. (2019). Current status of immune checkpoint inhibition in early-stage NSCLC. *Annals of Oncology*.

[30] Camidge D. R., Doebele R. C., Kerr K. M. (2019). Comparing and contrasting predictive biomarkers for immunotherapy and targeted therapy of NSCLC. *Nature Reviews. Clinical Oncology*.

[31] Proto C., Ferrara R., Signorelli D. (2019). Choosing wisely first line immunotherapy in non-small cell lung cancer (NSCLC): what to add and what to leave out. *Cancer Treatment Reviews*.

[32] Valentino F., Borra G., Allione P., Rossi L. (2018). Emerging targets in advanced non-small-cell lung cancer. *Future Oncology*.

[33] Baker S., Dahele M., Lagerwaard F. J., Senan S. (2016). A critical review of recent developments in radiotherapy for non-small cell lung cancer. *Radiation Oncology*.

[34] Wang Y., Gudikote J., Giri U. (2018). RAD50 expression is associated with poor clinical outcomes after radiotherapy for resected non-small cell lung cancer. *Clinical Cancer Research*.

[35] Wen J., Liu H., Wang L. (2018). Potentially Functional Variants of *ATG16L2* Predict Radiation Pneumonitis and Outcomes in Patients with Non -Small Cell Lung Cancer after Definitive Radiotherapy. *Journal of Thoracic Oncology*.

[36] Ding S., Li G., Dang J., Zhao X. (2018). The association of UNC5A expression with the clinicopathologic features and prognosis of radiotherapy in patients with non-small-cell lung cancer. *International Journal of Clinical and Experimental Pathology*.

[37] Corrigan A., Walker J. L., Wickramasinghe S. (2014). Pharmacogenetics of pemetrexed combination therapy in lung cancer: pathway analysis reveals novel toxicity associations. *The Pharmacogenomics Journal*.

[38] Xu R., Cao X. R., Zhang B. Q., Wang J. L., Wang L., Sun W. Q. (2019). BLACAT1 is negatively associated with prognosis in patients with NSCLC and inhibits cell progression, metastasis and epithelial-mesenchymal transition through down-regulating Wnt/*β*-catenin signaling pathway. *European Review for Medical and Pharmacological Sciences*.

[39] Bittel D. C., Kibiryeva N., Dasouki M., Knoll J. H., Butler M. G. (2006). A 9-year-old male with a duplication of chromosome 3p25.3p26.2: clinical report and gene expression analysis. *American Journal of Medical Genetics Part A*.

[40] Yuan K., Gao Z. J., Yuan W. D., Yuan J. Q., Wang Y. (2018). High expression of SLC6A10P contributes to poor prognosis in lung adenocarcinoma. *International Journal of Clinical and Experimental Pathology*.

[41] Ishiwata T., Matsuda Y., Yoshimura H. (2018). Pancreatic cancer stem cells: features and detection methods. *Pathology Oncology Research*.

[42] Yue Z., Li H. T., Yang Y. (2016). Identification of breast cancer candidate genes using gene co-expression and protein-protein interaction information. *Oncotarget*.

[43] von Eyben F. E. (2004). Chromosomes, genes, and development of testicular germ cell tumors. *Cancer Genetics and Cytogenetics*.

[44] Lefebvre C., Bachelot T., Filleron T. (2016). Mutational profile of metastatic breast cancers: a retrospective analysis. *PLoS Medicine*.

[45] Ayen A., Jimenez Martinez Y., Boulaiz H. (2020). Targeted gene delivery therapies for cervical cancer. *Cancers (Basel)*.

[46] Zhang X., Wan S., Yu Y. (2020). Identifying potential DNA methylation markers in early-stage colorectal cancer. *Genomics*.

[47] Li X., Tian R., Gao H. (2017). Identification of a histone family gene signature for predicting the prognosis of cervical cancer patients. *Scientific Reports*.

[48] Ganti A. K., Wang X., Stinchcombe T. E. (2019). Clinical prognostic model for older patients with advanced non-small cell lung cancer. *Journal of geriatric oncology*.

[49] Park W., Kwon D., Saravia D. (2018). Developing a predictive model for clinical outcomes of advanced non-small cell lung cancer patients treated with nivolumab. *Clinical Lung Cancer*.

[50] Van Laar R. K. (2012). Genomic signatures for predicting survival and adjuvant chemotherapy benefit in patients with non-small-cell lung cancer. *BMC Medical Genomics*.

[51] Ko E. C., Raben D., Formenti S. C. (2018). The integration of radiotherapy with immunotherapy for the treatment of non-small cell lung cancer. *Clinical Cancer Research*.

[52] Liu C., Hu Q., Hu K. (2019). Increased CD8+CD28+ T cells independently predict better early response to stereotactic ablative radiotherapy in patients with lung metastases from non-small cell lung cancer. *Journal of Translational Medicine*.

[53] Luo H., Ge H., Cui Y. (2018). Systemic inflammation biomarkers predict survival in patients of early stage non-small cell lung cancer treated with stereotactic ablative radiotherapy - a single center experience. *Journal of Cancer*.

[54] Liu C., Hu Q., Xu B. (2019). Peripheral memory and naïve T cells in non-small cell lung cancer patients with lung metastases undergoing stereotactic body radiotherapy: predictors of early tumor response. *Cancer Cell International*.

[55] Deng C., Wu S., Zhang L. (2018). Role of monocyte tissue factor on patients with non-small cell lung cancer. *The Clinical Respiratory Journal*.

[56] Han X., Guo B., Li Y., Zhu B. (2014). Tissue factor in tumor microenvironment: a systematic review. *Journal of Hematology & Oncology*.

[57] Liu C., Wu S., Liu G. (2017). Naive and memory T cells in patients with non-small cell lung cancer treated with radiotherapy and their prognostic value. *Chinese Journal of Clinical Oncology*.

