# Supplementary figures and images for: Identification and Prognostic Value Exploration of Radiotherapy Sensitivity-Associated Genes in Non-Small-Cell Lung Cancer
Source: Biomed Res Int. 2021 Sep 2;2021:5963868. doi: 10.1155/2021/5963868 (PMC8433590; doi:10.1155/2021/5963868)

Response 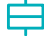 Disease\_progression 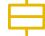 Partial\_response 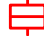 Complete\_response 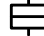 Stable\_disease

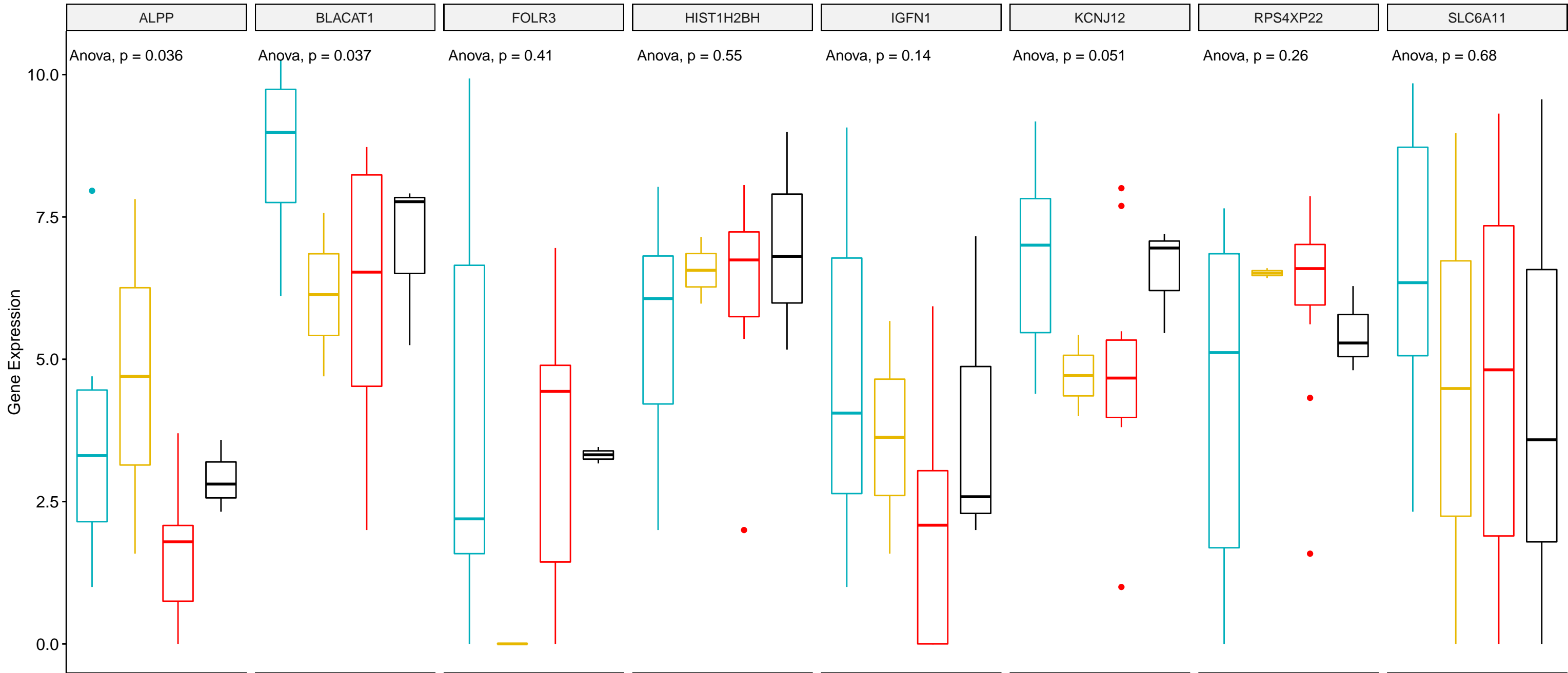

Supplement: Supplementary 1 — Figure S1: the expressions of FOLR3, SLC6A11, ALPP, IGFN1, KCNJ12, RPS4XP22, HIST1H2BH, and BLACAT1 in NSCLC samples with different radiotherapy response status. [file 5963868.f1.pdf]
