# Supplementary material for: Identification and Prognostic Value Exploration of Radiotherapy Sensitivity-Associated Genes in Non-Small-Cell Lung Cancer
Source: Biomed Res Int. 2021 Sep 2;2021:5963868. doi: 10.1155/2021/5963868 (PMC8433590; doi:10.1155/2021/5963868)
Supplement: Supplementary 2 — Table S1: clinical information of samples with radiotherapy response information. Table S2: genes in the red module. Table S3: the information of GO analysis based on the identified genes. Table S4: the information of KEGG analysis based on the identified genes. [file 5963868.f2.docx]

**Supplementary Material**

TableS1 Clinical information of samples with radiotherapy response information

| sample | Response | Age | Sex | New_tumor_event | Pathologic_M | Pathologic_N | Pathologic_T | Stage |
| --- | --- | --- | --- | --- | --- | --- | --- | --- |
| TCGA-33-AASI | Stable Disease | 65 | FEMALE | NO | MX | N1 | T2 | Stage IIB |
| TCGA-37-A5EL | Partial Response | 53 | MALE | YES | M0 | N0 | T3 | Stage IIB |
| TCGA-43-A475 | Complete Response | 67 | FEMALE | NO | M0 | N0 | T3 | Stage IIB |
| TCGA-52-7812 | Progressive Disease | 68 | MALE | YES | M0 | N2 | T2 | Stage IIIA |
| TCGA-56-7579 | Progressive Disease | 61 | MALE | YES | M0 | N1 | T3 | Stage IIIA |
| TCGA-56-7730 | Progressive Disease | 73 | MALE | NO | M0 | N0 | T2b | Stage IIA |
| TCGA-56-7822 | Progressive Disease | 75 | MALE | YES | M0 | N1 | T2b | Stage IIB |
| TCGA-56-8308 | Stable Disease | 79 | MALE | NO | MX | N0 | T3 | Stage IIB |
| TCGA-56-A4BY | Complete Response | 66 | MALE | NO | MX | N0 | T2a | Stage IB |
| TCGA-56-A62T | Complete Response | 78 | MALE | NO | MX | N0 | T2b | Stage IIA |
| TCGA-58-8391 | Complete Response | 57 | FEMALE | YES | M0 | N2 | T2 | Stage IIIA |
| TCGA-58-A46K | Complete Response | 59 | MALE | YES | M0 | N2 | T2 | Stage IIIA |
| TCGA-66-2757 | Progressive Disease | 65 | FEMALE | YES | M0 | N0 | T1 | Stage IA |
| TCGA-66-2759 | Complete Response | 66 | MALE | NO | M0 | N2 | T2 | Stage IIIA |
| TCGA-66-2778 | Complete Response | 68 | FEMALE | NO | M0 | N3 | T2 | Stage IIIB |
| TCGA-66-2783 | Complete Response | 67 | MALE | NO | M0 | N3 | T2 | Stage IIIB |
| TCGA-66-2794 | Complete Response | 64 | MALE | NO | M0 | N2 | T4 | Stage IIIB |
| TCGA-68-A59I | Stable Disease | 73 | FEMALE | YES | M0 | N1 | T3 | Stage IIIA |
| TCGA-70-6722 | Progressive Disease | 47 | MALE | YES | M0 | N1 | T3 | Stage IIIA |
| TCGA-70-6723 | Progressive Disease | 65 | MALE | YES | M0 | N0 | T3 | Stage IIA |
| TCGA-85-A4CN | Complete Response | 56 | FEMALE | NO | M0 | N1 | T2b | Stage IIB |
| TCGA-LA-A7SW | Progressive Disease | 71 | MALE | YES | MX | N1 | T3 | Stage IIIA |
| TCGA-NC-A5HF | Progressive Disease | 74 | MALE | YES | MX | N0 | T4 | Stage IIIB |
| TCGA-NC-A5HG | Complete Response | 59 | MALE | NO | M0 | N2 | T2 | Stage IIIA |
| TCGA-NC-A5HP | Progressive Disease | 69 | MALE | NO | M1b | N0 | T2a | Stage IV |
| TCGA-NC-A5HQ | Complete Response | 70 | MALE | NO | M0 | N2 | T3 | Stage IIIA |
| TCGA-NC-A5HT | Partial Response | 69 | MALE | NO | M0 | N1 | T3 | Stage IIIA |

TableS2 Genes in the red module

| Rank | GeneSymbol | Rank | GeneSymbol | Rank | GeneSymbol | Rank | GeneSymbol |
| --- | --- | --- | --- | --- | --- | --- | --- |
| 1 | MYH16 | 93 | WNT7A | 184 | AJAP1 | 275 | PCDHA11 |
| 2 | DPF1 | 94 | GOLGA7B | 185 | ZNF418 | 276 | AC226118.1 |
| 3 | STMN4 | 95 | GPR78 | 186 | RP11-88G17.6 | 277 | THEGL |
| 4 | MYO16 | 96 | MAGEC1 | 187 | SLC6A17 | 278 | YJEFN3 |
| 5 | MAGEC2 | 97 | AFF2 | 188 | RP11-366L20.2 | 279 | RP11-1E6.1 |
| 6 | LAMA3 | 98 | GLYATL2 | 189 | ZNF665 | 280 | RP11-230G5.2 |
| 7 | PTPRN | 99 | XDH | 190 | MFAP5 | 281 | LINC00589 |
| 8 | NPFFR2 | 100 | CDA | 191 | ZNF71 | 282 | RP11-962G15.1 |
| 9 | PKP2 | 101 | STC1 | 192 | ZNF560 | 283 | RP11-981G7.2 |
| 10 | LAMC2 | 102 | HIPK4 | 193 | ARC | 284 | PRSS51 |
| 11 | TRHDE | 103 | PDPN | 194 | RYR2 | 285 | LINC01151 |
| 12 | ADCY2 | 104 | LRRC38 | 195 | SMOC1 | 286 | ETV3L |
| 13 | HBQ1 | 105 | NTNG1 | 196 | APCDD1L | 287 | AF186192.1 |
| 14 | ZFHX4 | 106 | BRINP3 | 197 | L1CAM | 288 | RTL1 |
| 15 | IL11 | 107 | IL24 | 198 | POU3F3 | 289 | RP11-890B15.2 |
| 16 | NEFH | 108 | ALPP | 199 | LINC00632 | 290 | RP11-109L13.1 |
| 17 | APOL1 | 109 | IGFN1 | 200 | NEU4 | 291 | AF186192.5 |
| 18 | HNF4A | 110 | PTX3 | 201 | PSG5 | 292 | RP11-96B2.1 |
| 19 | NTSR1 | 111 | DNASE1L3 | 202 | CTC-425O23.2 | 293 | NOX5 |
| 20 | NKAIN4 | 112 | LRRC2 | 203 | KRTAP10-2 | 294 | DENND5B-AS1 |
| 21 | VGLL1 | 113 | ASB5 | 204 | RP11-497E19.1 | 295 | LINC00346 |
| 22 | IRG1 | 114 | SP8 | 205 | GAGE1 | 296 | RP11-771K4.1 |
| 23 | CALB1 | 115 | SUN3 | 206 | PCNPP3 | 297 | RP11-221N13.3 |
| 24 | ZFR2 | 116 | PHYHIPL | 207 | MAGEB1 | 298 | RP11-169D4.2 |
| 25 | HAS1 | 117 | RNASE7 | 208 | MEG3 | 299 | RP11-221N13.4 |
| 26 | DFNA5 | 118 | C10orf82 | 209 | PPIAP29 | 300 | RP11-817J15.2 |
| 27 | GCK | 119 | LDHC | 210 | RP1-97D16.1 | 301 | RP11-54A9.1 |
| 28 | ELAVL2 | 120 | ELFN2 | 211 | ZNF880 | 302 | RP11-498M15.1 |
| 29 | SORCS1 | 121 | TMEM92 | 212 | MEG9 | 303 | DIO3OS |
| 30 | CSF3 | 122 | ANKRD33 | 213 | LINC01208 | 304 | LINC00911 |
| 31 | CWH43 | 123 | NLRP7 | 214 | MIR503HG | 305 | CERS3-AS1 |
| 32 | FOLR3 | 124 | GGT6 | 215 | RP11-554I8.2 | 306 | RP4-597A16.2 |
| 33 | WNT5B | 125 | KLK4 | 216 | CITF22-49D8.1 | 307 | AGBL1-AS1 |
| 34 | KCNA1 | 126 | SOAT2 | 217 | CT62 | 308 | CTD-2311M21.2 |
| 35 | SLCO1B3 | 127 | MGAT5B | 218 | LINC00431 | 309 | RP11-800A3.4 |
| 36 | TREML2 | 128 | UGT3A2 | 219 | AC016582.2 | 310 | RP11-538I12.3 |
| 37 | SLC22A2 | 129 | IL1RAPL1 | 220 | RP11-112J3.16 | 311 | RP11-21B23.2 |
| 38 | SLC27A6 | 130 | COL22A1 | 221 | LINC01537 | 312 | RP11-244O19.1 |
| 39 | IL1A | 131 | CT55 | 222 | AC006042.6 | 313 | RP11-575H3.1 |
| 40 | RPE65 | 132 | CTNND2 | 223 | AP001271.3 | 314 | CTC-459F4.1 |
| 41 | NKX2-3 | 133 | FRMPD4 | 224 | FLJ43879 | 315 | CTD-2354A18.1 |
| 42 | PCDHB8 | 134 | IRX1 | 225 | RP11-400N13.2 | 316 | CTD-2311M21.3 |
| 43 | IL13RA2 | 135 | CDH2 | 226 | RP11-108M9.3 | 317 | GFY |
| 44 | ZNF137P | 136 | IRX2 | 227 | RP11-336A10.4 | 318 | LL22NC03-33B6.4 |
| 45 | KCNS1 | 137 | PSG6 | 228 | LINC00452 | 319 | RP11-1109M24.5 |
| 46 | TOX2 | 138 | C8orf74 | 229 | CTD-2554C21.3 | 320 | CTD-3162L10.1 |
| 47 | PAX8 | 139 | NPTX1 | 230 | AC007879.7 | 321 | ZNF790-AS1 |
| 48 | GRIA3 | 140 | ECEL1 | 231 | EMX2OS | 322 | CTD-2162K18.4 |
| 49 | NKX2-4 | 141 | WDR87 | 232 | NFE4 | 323 | RP11-879F14.2 |
| 50 | VGF | 142 | ADRA1D | 233 | PROX1-AS1 | 324 | ZNF571-AS1 |
| 51 | DPP6 | 143 | EPHX4 | 234 | RP4-738P15.1 | 325 | ZNF285 |
| 52 | UNC13A | 144 | AQPEP | 235 | AC005077.14 | 326 | CTD-2587H24.5 |
| 53 | GATA5 | 145 | DAB1 | 236 | RP11-775L16.1 | 327 | CTD-3220F14.1 |
| 54 | AKAP12 | 146 | TNFRSF10D | 237 | APCDD1L-AS1 | 328 | CTD-2554C21.2 |
| 55 | GFAP | 147 | C4orf26 | 238 | RP11-310H4.6 | 329 | ARL14EPL |
| 56 | ANO1 | 148 | SLC22A1 | 239 | RP11-336A10.5 | 330 | RP4-806M20.4 |
| 57 | ABHD12B | 149 | CIDEA | 240 | E2F6P4 | 331 | CT45A1 |
| 58 | RTBDN | 150 | FOXL1 | 241 | AC009502.1 | 332 | RP5-907D15.4 |
| 59 | SLC6A11 | 151 | TCERG1L | 242 | LINC00945 | 333 | ERVV-2 |
| 60 | CRP | 152 | FKBP9P1 | 243 | NALCN-AS1 | 334 | ZNF818P |
| 61 | SPX | 153 | HCG4 | 244 | RP11-398B16.2 | 335 | CTD-2245F17.3 |
| 62 | BAI3 | 154 | FBXO39 | 245 | LINC01271 | 336 | RP11-173C1.1 |
| 63 | NT5E | 155 | FLJ40194 | 246 | ACTBP12 | 337 | RP6-91H8.3 |
| 64 | TBR1 | 156 | ZBED2 | 247 | AK4P3 | 338 | RP11-394O4.5 |
| 65 | MMP20 | 157 | TMEM151A | 248 | RP11-100E13.1 | 339 | RP11-642C5.1 |
| 66 | BRDT | 158 | CITED4 | 249 | LINC00460 | 340 | BCRP9 |
| 67 | MYPN | 159 | TH | 250 | RP11-547D24.1 | 341 | RP11-350D17.3 |
| 68 | FGF5 | 160 | CTD-3064H18.4 | 251 | AC093326.3 | 342 | MMP28 |
| 69 | ERP27 | 161 | HLA-V | 252 | RP11-452D2.2 | 343 | RP11-324L17.1 |
| 70 | PLCZ1 | 162 | RGS7 | 253 | RP11-30P6.6 | 344 | RP11-344P13.6 |
| 71 | SYT16 | 163 | SOX1 | 254 | LINC00601 | 345 | RP5-1159O4.2 |
| 72 | DISP2 | 164 | RP1L1 | 255 | FAM103A2P | 346 | GRIN2B |
| 73 | ADAMTS18 | 165 | KCTD8 | 256 | LINC00941 | 347 | RP11-613E4.4 |
| 74 | MYOCD | 166 | KCNJ12 | 257 | MYO16-AS1 | 348 | RP3-333B15.5 |
| 75 | SLC14A1 | 167 | PCDH9 | 258 | TRHDE-AS1 | 349 | RP11-566K19.6 |
| 76 | SLC13A5 | 168 | WT1 | 259 | RP11-411K7.1 | 350 | RP11-65I12.1 |
| 77 | TINAGL1 | 169 | FLRT2 | 260 | LINC00383 | 351 | RP11-478J18.2 |
| 78 | FAM163A | 170 | NAT8L | 261 | RP13-143G15.4 | 352 | HIST1H2BH |
| 79 | LEFTY2 | 171 | LEMD1 | 262 | AC073257.2 | 353 | RP5-875H18.9 |
| 80 | PLEKHA6 | 172 | TRDN | 263 | AP004372.1 | 354 | HYDIN2 |
| 81 | 43894 | 173 | CYP27C1 | 264 | MKRN4P | 355 | RP11-88E10.4 |
| 82 | MYH15 | 174 | ZNF732 | 265 | RPS4XP22 | 356 | KB-68A7.2 |
| 83 | PNLDC1 | 175 | CCK | 266 | L1TD1 | 357 | CH17-13I23.3 |
| 84 | VIP | 176 | DNER | 267 | RPSAP52 | 358 | RP11-677M24.1 |
| 85 | RGS20 | 177 | EYS | 268 | RP13-463N16.6 | 359 | RP11-102K13.5 |
| 86 | HMGA2 | 178 | INSC | 269 | PEG10 | 360 | AC253572.1 |
| 87 | THRB | 179 | SPRED3 | 270 | CCDC169 | 361 | RP4-737E23.5 |
| 88 | KCNJ1 | 180 | RP11-159F24.2 | 271 | RP11-7F17.1 | 362 | CH507-154B10.1 |
| 89 | RBM46 | 181 | GAGE2A | 272 | LINC01322 | 363 | RP11-486M23.3 |
| 90 | PTH | 182 | VSTM1 | 273 | RP11-1094H24.4 | 364 | ELDR |
| 91 | PTPRR | 183 | FAM150B | 274 | RP11-94H18.1 | 365 | BLACAT1 |
| 92 | CDH12 |  |  |  |  |  |  |

Table S3. The information of GO analysis based on the identified genes

| ,"ONTOLOGY","ID","Description","GeneRatio","BgRatio","pvalue","p.adjust","qvalue","geneID","Count" |
| --- |
| GO:0050808,"BP","GO:0050808","synapse organization","12/183","304/17653",8.11909611776156e-05,0.0885964933451525,0.0807864927405441,"PCDHB8/UNC13A/WNT7A/IL1RAPL1/CTNND2/FRMPD4/CDH2/FLRT2/DNER/ARC/L1CAM/GRIN2B",12 |
| GO:0072207,"BP","GO:0072207","metanephric epithelium development","4/183","27/17653",0.000162747841380891,0.0885964933451525,0.0807864927405441,"CALB1/PAX8/WT1/POU3F3",4 |
| GO:0072070,"BP","GO:0072070","loop of Henle development","3/183","11/17653",0.000170086876620251,0.0885964933451525,0.0807864927405441,"IRX1/IRX2/POU3F3",3 |
| GO:0010644,"BP","GO:0010644","cell communication by electrical coupling","4/183","28/17653",0.000188342571731697,0.0885964933451525,0.0807864927405441,"PKP2/KCNA1/TRDN/RYR2",4 |
| GO:0072017,"BP","GO:0072017","distal tubule development","3/183","12/17653",0.000225054456304738,0.0885964933451525,0.0807864927405441,"CALB1/PAX8/POU3F3",3 |
| GO:0006836,"BP","GO:0006836","neurotransmitter transport","10/183","246/17653",0.000253294945824623,0.0885964933451525,0.0807864927405441,"NTSR1/SLC22A2/UNC13A/GFAP/SLC6A11/SYT16/WNT7A/SLC22A1/TH/SLC6A17",10 |
| GO:0032414,"BP","GO:0032414","positive regulation of ion transmembrane transporter activity","6/183","88/17653",0.000306490107935272,0.0885964933451525,0.0807864927405441,"NTSR1/KCNA1/LRRC38/TRDN/ARC/RYR2",6 |
| GO:0001656,"BP","GO:0001656","metanephros development","6/183","89/17653",0.000325854251605917,0.0885964933451525,0.0807864927405441,"CALB1/PAX8/IRX1/IRX2/WT1/POU3F3",6 |
| GO:2001259,"BP","GO:2001259","positive regulation of cation channel activity","5/183","58/17653",0.000332879174794656,0.0885964933451525,0.0807864927405441,"NTSR1/KCNA1/LRRC38/TRDN/ARC",5 |
| GO:0072080,"BP","GO:0072080","nephron tubule development","6/183","92/17653",0.000389750908000549,0.0885964933451525,0.0807864927405441,"CALB1/PAX8/IRX1/IRX2/WT1/POU3F3",6 |
| GO:0010959,"BP","GO:0010959","regulation of metal ion transport","12/183","363/17653",0.000417234491111548,0.0885964933451525,0.0807864927405441,"PKP2/NTSR1/NKAIN4/GCK/KCNA1/KCNS1/DPP6/STC1/LRRC38/ADRA1D/TRDN/RYR2",12 |
| GO:0061326,"BP","GO:0061326","renal tubule development","6/183","94/17653",0.000437513547383469,0.0885964933451525,0.0807864927405441,"CALB1/PAX8/IRX1/IRX2/WT1/POU3F3",6 |
| GO:0032411,"BP","GO:0032411","positive regulation of transporter activity","6/183","98/17653",0.000546664348491627,0.0964405064575798,0.0879390366442302,"NTSR1/KCNA1/LRRC38/TRDN/ARC/RYR2",6 |
| GO:0051284,"BP","GO:0051284","positive regulation of sequestering of calcium ion","3/183","16/17653",0.000555624317039554,0.0964405064575798,0.0879390366442302,"NTSR1/TRDN/RYR2",3 |
| GO:0043266,"BP","GO:0043266","regulation of potassium ion transport","6/183","101/17653",0.000641576044460509,0.100250006240129,0.0914127195735202,"GCK/KCNA1/KCNS1/DPP6/LRRC38/ADRA1D",6 |
| GO:0050804,"BP","GO:0050804","modulation of chemical synaptic transmission","11/183","335/17653",0.000765098244654521,0.100250006240129,0.0914127195735202,"NTSR1/CALB1/GRIA3/VGF/UNC13A/GFAP/WNT7A/CDH2/ADRA1D/ARC/GRIN2B",11 |
| GO:0099177,"BP","GO:0099177","regulation of trans-synaptic signaling","11/183","335/17653",0.000765098244654521,0.100250006240129,0.0914127195735202,"NTSR1/CALB1/GRIA3/VGF/UNC13A/GFAP/WNT7A/CDH2/ADRA1D/ARC/GRIN2B",11 |
| GO:0050806,"BP","GO:0050806","positive regulation of synaptic transmission","7/183","146/17653",0.000829276361236628,0.100250006240129,0.0914127195735202,"NTSR1/CALB1/UNC13A/GFAP/WNT7A/ARC/GRIN2B",7 |
| GO:0072210,"BP","GO:0072210","metanephric nephron development","4/183","41/17653",0.000838745946001645,0.100250006240129,0.0914127195735202,"PAX8/IRX1/IRX2/WT1",4 |
| GO:0043270,"BP","GO:0043270","positive regulation of ion transport","9/183","240/17653",0.000915947104912251,0.100250006240129,0.0914127195735202,"PKP2/NTSR1/KCNA1/STC1/LRRC38/TRDN/CCK/ARC/RYR2",9 |
| GO:0072079,"BP","GO:0072079","nephron tubule formation","3/183","19/17653",0.000939680214699637,0.100250006240129,0.0914127195735202,"PAX8/IRX1/IRX2",3 |
| GO:0001654,"BP","GO:0001654","eye development","11/183","344/17653",0.00094952794466252,0.100250006240129,0.0914127195735202,"CALB1/WNT5B/RPE65/ADAMTS18/MYH15/WNT7A/TH/SOX1/RP1L1/WT1/SMOC1",11 |
| GO:0072009,"BP","GO:0072009","nephron epithelium development","6/183","109/17653",0.00095769333517699,0.100250006240129,0.0914127195735202,"CALB1/PAX8/IRX1/IRX2/WT1/POU3F3",6 |
| GO:0015893,"BP","GO:0015893","drug transport","8/183","195/17653",0.000990123518421026,0.100250006240129,0.0914127195735202,"HBQ1/NTSR1/FOLR3/SLC22A2/SLC13A5/ADRA1D/SLC22A1/SLC6A17",8 |
| GO:0072234,"BP","GO:0072234","metanephric nephron tubule development","3/183","20/17653",0.00109711482974394,0.106639561451111,0.0972390196467788,"CALB1/PAX8/POU3F3",3 |
| GO:1903115,"BP","GO:1903115","regulation of actin filament-based movement","4/183","45/17653",0.00119491351018947,0.111678454990785,0.101833722224649,"PKP2/STC1/PDPN/RYR2",4 |
| GO:1904062,"BP","GO:1904062","regulation of cation transmembrane transport","10/183","302/17653",0.00124155370662853,0.111739833596568,0.101889690154115,"NTSR1/KCNA1/KCNS1/DPP6/LRRC38/ADRA1D/TRDN/ARC/RYR2/NOX5",10 |
| GO:2001257,"BP","GO:2001257","regulation of cation channel activity","7/183","159/17653",0.00136320432042242,0.118306660665232,0.107877635131173,"NTSR1/KCNA1/KCNS1/LRRC38/ADRA1D/TRDN/ARC",7 |
| GO:0045109,"BP","GO:0045109","intermediate filament organization","3/183","22/17653",0.00145966999392733,0.120500357148415,0.109877951829072,"PKP2/NEFH/GFAP",3 |
| GO:0072170,"BP","GO:0072170","metanephric tubule development","3/183","23/17653",0.00166589768406184,0.120500357148415,0.109877951829072,"CALB1/PAX8/POU3F3",3 |
| GO:0072243,"BP","GO:0072243","metanephric nephron epithelium development","3/183","23/17653",0.00166589768406184,0.120500357148415,0.109877951829072,"CALB1/PAX8/POU3F3",3 |
| GO:0086064,"BP","GO:0086064","cell communication by electrical coupling involved in cardiac conduction","3/183","23/17653",0.00166589768406184,0.120500357148415,0.109877951829072,"PKP2/TRDN/RYR2",3 |
| GO:0006937,"BP","GO:0006937","regulation of muscle contraction","7/183","165/17653",0.00168601322759099,0.120500357148415,0.109877951829072,"PKP2/KCNA1/SPX/MYOCD/STC1/ADRA1D/RYR2",7 |
| GO:0050807,"BP","GO:0050807","regulation of synapse organization","7/183","165/17653",0.00168601322759099,0.120500357148415,0.109877951829072,"WNT7A/IL1RAPL1/FRMPD4/CDH2/FLRT2/ARC/GRIN2B",7 |
| GO:1904064,"BP","GO:1904064","positive regulation of cation transmembrane transport","6/183","124/17653",0.00185930935858731,0.12908919261049,0.117709660145152,"NTSR1/KCNA1/LRRC38/TRDN/ARC/RYR2",6 |
| GO:0023061,"BP","GO:0023061","signal release","12/183","435/17653",0.00198440082883613,0.133947055946439,0.122139290780703,"PTPRN/IL11/HNF4A/GCK/SLC22A2/PAX8/VGF/UNC13A/ANO1/SYT16/VIP/WNT7A",12 |
| GO:0050803,"BP","GO:0050803","regulation of synapse structure or activity","7/183","176/17653",0.00242937626615801,0.155844407711104,0.14210633668264,"WNT7A/IL1RAPL1/FRMPD4/CDH2/FLRT2/ARC/GRIN2B",7 |
| GO:0034765,"BP","GO:0034765","regulation of ion transmembrane transport","12/183","446/17653",0.00243707304239587,0.155844407711104,0.14210633668264,"NTSR1/KCNA1/KCNS1/DPP6/KCNJ1/LRRC38/ADRA1D/KCNJ12/TRDN/ARC/RYR2/NOX5",12 |
| GO:0009582,"BP","GO:0009582","detection of abiotic stimulus","6/183","132/17653",0.00254743717255767,0.158724931520901,0.144732935175004,"NPFFR2/NTSR1/KCNA1/RPE65/ANO1/EYS",6 |
| GO:0060993,"BP","GO:0060993","kidney morphogenesis","5/183","92/17653",0.00269265976009701,0.160636275163206,0.146475789135174,"CALB1/PAX8/IRX1/IRX2/WT1",5 |
| GO:0043062,"BP","GO:0043062","extracellular structure organization","11/183","395/17653",0.00282088500508917,0.160636275163206,0.146475789135174,"LAMA3/LAMC2/HAS1/GFAP/MMP20/PDPN/KLK4/SOAT2/WT1/FLRT2/MFAP5",11 |
| GO:0034767,"BP","GO:0034767","positive regulation of ion transmembrane transport","6/183","136/17653",0.00295584640689431,0.160636275163206,0.146475789135174,"NTSR1/KCNA1/LRRC38/TRDN/ARC/RYR2",6 |
| GO:0022617,"BP","GO:0022617","extracellular matrix disassembly","5/183","94/17653",0.00295615063286451,0.160636275163206,0.146475789135174,"LAMA3/LAMC2/MMP20/PDPN/KLK4",5 |
| GO:0030198,"BP","GO:0030198","extracellular matrix organization","10/183","341/17653",0.00301575175426134,0.160636275163206,0.146475789135174,"LAMA3/LAMC2/HAS1/GFAP/MMP20/PDPN/KLK4/WT1/FLRT2/MFAP5",10 |
| GO:0072006,"BP","GO:0072006","nephron development","6/183","137/17653",0.00306518592074182,0.160636275163206,0.146475789135174,"CALB1/PAX8/IRX1/IRX2/WT1/POU3F3",6 |
| GO:0019233,"BP","GO:0019233","sensory perception of pain","5/183","95/17653",0.00309465646774103,0.160636275163206,0.146475789135174,"NTSR1/KCNA1/ANO1/SPX/CCK",5 |
| GO:0032412,"BP","GO:0032412","regulation of ion transmembrane transporter activity","8/183","234/17653",0.00310695676241593,0.160636275163206,0.146475789135174,"NTSR1/KCNA1/KCNS1/LRRC38/ADRA1D/TRDN/ARC/RYR2",8 |
| GO:1903522,"BP","GO:1903522","regulation of blood circulation","9/183","290/17653",0.00332981104123841,0.165792838885447,0.1511777889772,"PKP2/SPX/THRB/STC1/ADRA1D/TH/KCNJ12/TRDN/RYR2",9 |
| GO:0034764,"BP","GO:0034764","positive regulation of transmembrane transport","7/183","187/17653",0.00340307460358049,0.165792838885447,0.1511777889772,"NTSR1/KCNA1/PTH/LRRC38/TRDN/ARC/RYR2",7 |
| GO:0072073,"BP","GO:0072073","kidney epithelium development","6/183","140/17653",0.00341137528570879,0.165792838885447,0.1511777889772,"CALB1/PAX8/IRX1/IRX2/WT1/POU3F3",6 |
| GO:0022898,"BP","GO:0022898","regulation of transmembrane transporter activity","8/183","240/17653",0.00362240844029967,0.172597108037808,0.157382244929428,"NTSR1/KCNA1/KCNS1/LRRC38/ADRA1D/TRDN/ARC/RYR2",8 |
| GO:0006813,"BP","GO:0006813","potassium ion transport","8/183","242/17653",0.003808191729041,0.175671279247285,0.160185420322952,"GCK/KCNA1/KCNS1/DPP6/KCNJ1/LRRC38/ADRA1D/KCNJ12",8 |
| GO:0043010,"BP","GO:0043010","camera-type eye development","9/183","297/17653",0.00389334521998354,0.175671279247285,0.160185420322952,"CALB1/WNT5B/RPE65/MYH15/WNT7A/TH/SOX1/RP1L1/WT1",9 |
| GO:0008016,"BP","GO:0008016","regulation of heart contraction","8/183","243/17653",0.00390380620549522,0.175671279247285,0.160185420322952,"PKP2/SPX/THRB/STC1/TH/KCNJ12/TRDN/RYR2",8 |
| GO:0099173,"BP","GO:0099173","postsynapse organization","5/183","102/17653",0.00419845816058515,0.184250430979502,0.168008298553975,"WNT7A/CTNND2/CDH2/ARC/GRIN2B",5 |
| GO:0009954,"BP","GO:0009954","proximal/distal pattern formation","3/183","32/17653",0.00435760137650362,0.184250430979502,0.168008298553975,"SP8/IRX1/IRX2",3 |
| GO:0007588,"BP","GO:0007588","excretion","4/183","64/17653",0.00437541998769887,0.184250430979502,0.168008298553975,"SPX/KCNJ1/STC1/POU3F3",4 |
| GO:0071838,"BP","GO:0071838","cell proliferation in bone marrow","2/183","10/17653",0.00455363402234774,0.184250430979502,0.168008298553975,"HMGA2/PTH",2 |
| GO:0071863,"BP","GO:0071863","regulation of cell proliferation in bone marrow","2/183","10/17653",0.00455363402234774,0.184250430979502,0.168008298553975,"HMGA2/PTH",2 |
| GO:0007189,"BP","GO:0007189","adenylate cyclase-activating G-protein coupled receptor signaling pathway","5/183","104/17653",0.00455956843218807,0.184250430979502,0.168008298553975,"ADCY2/VIP/PTH/GPR78/ADRA1D",5 |
| GO:0051926,"BP","GO:0051926","negative regulation of calcium ion transport","4/183","65/17653",0.00462521658014387,0.184250430979502,0.168008298553975,"NTSR1/STC1/ADRA1D/TRDN",4 |
| GO:0003338,"BP","GO:0003338","metanephros morphogenesis","3/183","33/17653",0.00475721144792801,0.185116707323242,0.16879821048968,"CALB1/PAX8/WT1",3 |
| GO:0015849,"BP","GO:0015849","organic acid transport","9/183","309/17653",0.00503075993363326,0.185116707323242,0.16879821048968,"NTSR1/FOLR3/SLCO1B3/SLC27A6/SLC6A11/SPX/SLC13A5/CCK/SLC6A17",9 |
| GO:0046942,"BP","GO:0046942","carboxylic acid transport","9/183","309/17653",0.00503075993363326,0.185116707323242,0.16879821048968,"NTSR1/FOLR3/SLCO1B3/SLC27A6/SLC6A11/SPX/SLC13A5/CCK/SLC6A17",9 |
| GO:0045987,"BP","GO:0045987","positive regulation of smooth muscle contraction","3/183","34/17653",0.00517825715900809,0.185116707323242,0.16879821048968,"SPX/MYOCD/ADRA1D",3 |
| GO:0051930,"BP","GO:0051930","regulation of sensory perception of pain","3/183","34/17653",0.00517825715900809,0.185116707323242,0.16879821048968,"NTSR1/SPX/CCK",3 |
| GO:0051931,"BP","GO:0051931","regulation of sensory perception","3/183","34/17653",0.00517825715900809,0.185116707323242,0.16879821048968,"NTSR1/SPX/CCK",3 |
| GO:0032409,"BP","GO:0032409","regulation of transporter activity","8/183","255/17653",0.00520199185935191,0.185116707323242,0.16879821048968,"NTSR1/KCNA1/KCNS1/LRRC38/ADRA1D/TRDN/ARC/RYR2",8 |
| GO:0007416,"BP","GO:0007416","synapse assembly","6/183","153/17653",0.00525640033140071,0.185116707323242,0.16879821048968,"PCDHB8/WNT7A/IL1RAPL1/CDH2/FLRT2/DNER",6 |
| GO:0007494,"BP","GO:0007494","midgut development","2/183","11/17653",0.00552775561028719,0.187590928158384,0.171054322622222,"DAB1/FOXL1",2 |
| GO:0031581,"BP","GO:0031581","hemidesmosome assembly","2/183","11/17653",0.00552775561028719,0.187590928158384,0.171054322622222,"LAMA3/LAMC2",2 |
| GO:0021987,"BP","GO:0021987","cerebral cortex development","5/183","109/17653",0.00555824972321138,0.187590928158384,0.171054322622222,"TBR1/CDH2/DAB1/TH/POU3F3",5 |
| GO:0097061,"BP","GO:0097061","dendritic spine organization","4/183","70/17653",0.00601865313935115,0.195238523095528,0.178027763099886,"WNT7A/CTNND2/ARC/GRIN2B",4 |
| GO:0009914,"BP","GO:0009914","hormone transport","9/183","318/17653",0.00604158734997446,0.195238523095528,0.178027763099886,"PTPRN/IL11/HNF4A/GCK/PAX8/VGF/ANO1/VIP/SLC22A1",9 |
| GO:0006171,"BP","GO:0006171","cAMP biosynthetic process","4/183","71/17653",0.0063273063089198,0.195238523095528,0.178027763099886,"NPFFR2/ADCY2/PTH/ADRA1D",4 |
| GO:0043252,"BP","GO:0043252","sodium-independent organic anion transport","3/183","37/17653",0.00657301886944229,0.195238523095528,0.178027763099886,"SLCO1B3/SLC22A2/SLC22A1",3 |
| GO:0008228,"BP","GO:0008228","opsonization","2/183","12/17653",0.00658829584108368,0.195238523095528,0.178027763099886,"CRP/PTX3",2 |
| GO:0010623,"BP","GO:0010623","programmed cell death involved in cell development","2/183","12/17653",0.00658829584108368,0.195238523095528,0.178027763099886,"IL1A/DNASE1L3",2 |
| GO:0015740,"BP","GO:0015740","C4-dicarboxylate transport","2/183","12/17653",0.00658829584108368,0.195238523095528,0.178027763099886,"NTSR1/SLC13A5",2 |
| GO:0032096,"BP","GO:0032096","negative regulation of response to food","2/183","12/17653",0.00658829584108368,0.195238523095528,0.178027763099886,"SPX/CCK",2 |
| GO:0032099,"BP","GO:0032099","negative regulation of appetite","2/183","12/17653",0.00658829584108368,0.195238523095528,0.178027763099886,"SPX/CCK",2 |
| GO:0044793,"BP","GO:0044793","negative regulation by host of viral process","2/183","12/17653",0.00658829584108368,0.195238523095528,0.178027763099886,"CRP/PTX3",2 |
| GO:0060291,"BP","GO:0060291","long-term synaptic potentiation","4/183","73/17653",0.00697576488881375,0.196176651233695,0.178883192916148,"CALB1/GFAP/ARC/GRIN2B",4 |
| GO:0072078,"BP","GO:0072078","nephron tubule morphogenesis","4/183","73/17653",0.00697576488881375,0.196176651233695,0.178883192916148,"PAX8/IRX1/IRX2/WT1",4 |
| GO:0106027,"BP","GO:0106027","neuron projection organization","4/183","73/17653",0.00697576488881375,0.196176651233695,0.178883192916148,"WNT7A/CTNND2/ARC/GRIN2B",4 |
| GO:0021543,"BP","GO:0021543","pallium development","6/183","163/17653",0.00710797170066946,0.196176651233695,0.178883192916148,"KCNA1/TBR1/CDH2/DAB1/TH/POU3F3",6 |
| GO:0072088,"BP","GO:0072088","nephron epithelium morphogenesis","4/183","75/17653",0.00766670765012695,0.196176651233695,0.178883192916148,"PAX8/IRX1/IRX2/WT1",4 |
| GO:0015697,"BP","GO:0015697","quaternary ammonium group transport","2/183","13/17653",0.0077333782635215,0.196176651233695,0.178883192916148,"SLC22A2/SLC22A1",2 |
| GO:0015812,"BP","GO:0015812","gamma-aminobutyric acid transport","2/183","13/17653",0.0077333782635215,0.196176651233695,0.178883192916148,"NTSR1/SLC6A11",2 |
| GO:0042574,"BP","GO:0042574","retinal metabolic process","2/183","13/17653",0.0077333782635215,0.196176651233695,0.178883192916148,"RPE65/CYP27C1",2 |
| GO:0060732,"BP","GO:0060732","positive regulation of inositol phosphate biosynthetic process","2/183","13/17653",0.0077333782635215,0.196176651233695,0.178883192916148,"NTSR1/PTH",2 |
| GO:0070307,"BP","GO:0070307","lens fiber cell development","2/183","13/17653",0.0077333782635215,0.196176651233695,0.178883192916148,"WNT5B/WNT7A",2 |
| GO:0072044,"BP","GO:0072044","collecting duct development","2/183","13/17653",0.0077333782635215,0.196176651233695,0.178883192916148,"CALB1/PAX8",2 |
| GO:0003002,"BP","GO:0003002","regionalization","9/183","333/17653",0.00806669469909253,0.196176651233695,0.178883192916148,"PAX8/TBR1/WNT7A/SP8/IRX1/IRX2/SOX1/WT1/ARC",9 |
| GO:0010769,"BP","GO:0010769","regulation of cell morphogenesis involved in differentiation","8/183","275/17653",0.00806989718767354,0.196176651233695,0.178883192916148,"TBR1/WNT7A/PDPN/IL1RAPL1/CDH2/DAB1/ARC/L1CAM",8 |
| GO:0060047,"BP","GO:0060047","heart contraction","8/183","275/17653",0.00806989718767354,0.196176651233695,0.178883192916148,"PKP2/SPX/THRB/STC1/TH/KCNJ12/TRDN/RYR2",8 |
| GO:0007409,"BP","GO:0007409","axonogenesis","11/183","456/17653",0.00813175213069951,0.196176651233695,0.178883192916148,"NEFH/TBR1/WNT7A/LRRC38/NTNG1/CDH2/NPTX1/DAB1/FLRT2/CCK/L1CAM",11 |
| GO:0001659,"BP","GO:0001659","temperature homeostasis","3/183","40/17653",0.00817085946886843,0.196176651233695,0.178883192916148,"NTSR1/IL1A/CIDEA",3 |
| GO:0086004,"BP","GO:0086004","regulation of cardiac muscle cell contraction","3/183","40/17653",0.00817085946886843,0.196176651233695,0.178883192916148,"PKP2/STC1/RYR2",3 |
| GO:0048167,"BP","GO:0048167","regulation of synaptic plasticity","6/183","168/17653",0.00819260333846577,0.196176651233695,0.178883192916148,"CALB1/VGF/UNC13A/GFAP/ARC/GRIN2B",6 |
| GO:0061333,"BP","GO:0061333","renal tubule morphogenesis","4/183","77/17653",0.00840123648743545,0.196176651233695,0.178883192916148,"PAX8/IRX1/IRX2/WT1",4 |
| GO:0072028,"BP","GO:0072028","nephron morphogenesis","4/183","77/17653",0.00840123648743545,0.196176651233695,0.178883192916148,"PAX8/IRX1/IRX2/WT1",4 |
| GO:0003015,"BP","GO:0003015","heart process","8/183","278/17653",0.00858598959284514,0.196176651233695,0.178883192916148,"PKP2/SPX/THRB/STC1/TH/KCNJ12/TRDN/RYR2",8 |
| GO:0015711,"BP","GO:0015711","organic anion transport","11/183","460/17653",0.00865022632712864,0.196176651233695,0.178883192916148,"NTSR1/FOLR3/SLCO1B3/SLC22A2/SLC27A6/SLC6A11/SPX/SLC13A5/SLC22A1/CCK/SLC6A17",11 |
| GO:0001570,"BP","GO:0001570","vasculogenesis","4/183","78/17653",0.00878517869098056,0.196176651233695,0.178883192916148,"MYOCD/WNT7A/XDH/WT1",4 |
| GO:0006855,"BP","GO:0006855","drug transmembrane transport","4/183","78/17653",0.00878517869098056,0.196176651233695,0.178883192916148,"NTSR1/SLC22A2/SLC13A5/SLC22A1",4 |
| GO:0014048,"BP","GO:0014048","regulation of glutamate secretion","2/183","14/17653",0.00896115567363791,0.196176651233695,0.178883192916148,"NTSR1/CCK",2 |
| GO:0032105,"BP","GO:0032105","negative regulation of response to extracellular stimulus","2/183","14/17653",0.00896115567363791,0.196176651233695,0.178883192916148,"SPX/CCK",2 |
| GO:0032108,"BP","GO:0032108","negative regulation of response to nutrient levels","2/183","14/17653",0.00896115567363791,0.196176651233695,0.178883192916148,"SPX/CCK",2 |
| GO:0072216,"BP","GO:0072216","positive regulation of metanephros development","2/183","14/17653",0.00896115567363791,0.196176651233695,0.178883192916148,"PAX8/WT1",2 |
| GO:0072488,"BP","GO:0072488","ammonium transmembrane transport","2/183","14/17653",0.00896115567363791,0.196176651233695,0.178883192916148,"SLC22A2/SLC22A1",2 |
| GO:0015872,"BP","GO:0015872","dopamine transport","3/183","42/17653",0.00935202685375089,0.20110995800544,0.183381616461534,"SLC22A2/ADRA1D/SLC22A1",3 |
| GO:0042461,"BP","GO:0042461","photoreceptor cell development","3/183","42/17653",0.00935202685375089,0.20110995800544,0.183381616461534,"RPE65/TH/RP1L1",3 |
| GO:0045104,"BP","GO:0045104","intermediate filament cytoskeleton organization","3/183","43/17653",0.00997799497100568,0.202891362670704,0.185005985887733,"PKP2/NEFH/GFAP",3 |
| GO:1903170,"BP","GO:1903170","negative regulation of calcium ion transmembrane transport","3/183","43/17653",0.00997799497100568,0.202891362670704,0.185005985887733,"NTSR1/ADRA1D/TRDN",3 |
| GO:1901379,"BP","GO:1901379","regulation of potassium ion transmembrane transport","4/183","81/17653",0.0100052513011682,0.202891362670704,0.185005985887733,"KCNA1/KCNS1/DPP6/LRRC38",4 |
| GO:0010919,"BP","GO:0010919","regulation of inositol phosphate biosynthetic process","2/183","15/17653",0.0102698097154307,0.202891362670704,0.185005985887733,"NTSR1/PTH",2 |
| GO:0045725,"BP","GO:0045725","positive regulation of glycogen biosynthetic process","2/183","15/17653",0.0102698097154307,0.202891362670704,0.185005985887733,"GCK/PTH",2 |
| GO:0050961,"BP","GO:0050961","detection of temperature stimulus involved in sensory perception","2/183","15/17653",0.0102698097154307,0.202891362670704,0.185005985887733,"NTSR1/ANO1",2 |
| GO:0050965,"BP","GO:0050965","detection of temperature stimulus involved in sensory perception of pain","2/183","15/17653",0.0102698097154307,0.202891362670704,0.185005985887733,"NTSR1/ANO1",2 |
| GO:0051280,"BP","GO:0051280","negative regulation of release of sequestered calcium ion into cytosol","2/183","15/17653",0.0102698097154307,0.202891362670704,0.185005985887733,"NTSR1/TRDN",2 |
| GO:0051957,"BP","GO:0051957","positive regulation of amino acid transport","2/183","15/17653",0.0102698097154307,0.202891362670704,0.185005985887733,"NTSR1/CCK",2 |
| GO:1903818,"BP","GO:1903818","positive regulation of voltage-gated potassium channel activity","2/183","15/17653",0.0102698097154307,0.202891362670704,0.185005985887733,"KCNA1/LRRC38",2 |
| GO:0045103,"BP","GO:0045103","intermediate filament-based process","3/183","44/17653",0.010627787694377,0.208270355623678,0.189910807272186,"PKP2/NEFH/GFAP",3 |
| GO:0009581,"BP","GO:0009581","detection of external stimulus","5/183","129/17653",0.0111078615131566,0.212672503448077,0.19392489484869,"NTSR1/KCNA1/RPE65/ANO1/EYS",5 |
| GO:0015837,"BP","GO:0015837","amine transport","4/183","84/17653",0.0113302512211332,0.212672503448077,0.19392489484869,"NTSR1/ADRA1D/TH/CCK",4 |
| GO:0006936,"BP","GO:0006936","muscle contraction","9/183","352/17653",0.0113307172543123,0.212672503448077,0.19392489484869,"PKP2/KCNA1/SPX/MYOCD/STC1/ADRA1D/KCNJ12/TRDN/RYR2",9 |
| GO:0007379,"BP","GO:0007379","segment specification","2/183","16/17653",0.0116575504866294,0.212672503448077,0.19392489484869,"IRX1/IRX2",2 |
| GO:0035988,"BP","GO:0035988","chondrocyte proliferation","2/183","16/17653",0.0116575504866294,0.212672503448077,0.19392489484869,"HMGA2/STC1",2 |
| GO:0048535,"BP","GO:0048535","lymph node development","2/183","16/17653",0.0116575504866294,0.212672503448077,0.19392489484869,"NKX2-3/PDPN",2 |
| GO:0050908,"BP","GO:0050908","detection of light stimulus involved in visual perception","2/183","16/17653",0.0116575504866294,0.212672503448077,0.19392489484869,"RPE65/EYS",2 |
| GO:0050962,"BP","GO:0050962","detection of light stimulus involved in sensory perception","2/183","16/17653",0.0116575504866294,0.212672503448077,0.19392489484869,"RPE65/EYS",2 |
| GO:0070875,"BP","GO:0070875","positive regulation of glycogen metabolic process","2/183","16/17653",0.0116575504866294,0.212672503448077,0.19392489484869,"GCK/PTH",2 |
| GO:0007215,"BP","GO:0007215","glutamate receptor signaling pathway","4/183","85/17653",0.0117957779473811,0.212672503448077,0.19392489484869,"GRIA3/UNC13A/ARC/GRIN2B",4 |
| GO:0045471,"BP","GO:0045471","response to ethanol","5/183","131/17653",0.0118151390804487,0.212672503448077,0.19392489484869,"CSF3/PTH/ADRA1D/TH/GRIN2B",5 |
| GO:0048565,"BP","GO:0048565","digestive tract development","5/183","133/17653",0.0125524589403403,0.222335991656326,0.202736522606266,"NKX2-3/GATA5/MYOCD/DAB1/FOXL1",5 |
| GO:0099504,"BP","GO:0099504","synaptic vesicle cycle","5/183","133/17653",0.0125524589403403,0.222335991656326,0.202736522606266,"UNC13A/SYT16/WNT7A/CDH2/TH",5 |
| GO:0007611,"BP","GO:0007611","learning or memory","7/183","240/17653",0.0127238482568054,0.222335991656326,0.202736522606266,"NTSR1/CALB1/TBR1/AFF2/TH/ARC/GRIN2B",7 |
| GO:0006835,"BP","GO:0006835","dicarboxylic acid transport","4/183","87/17653",0.0127632820572192,0.222335991656326,0.202736522606266,"NTSR1/FOLR3/SLC13A5/CCK",4 |
| GO:0007342,"BP","GO:0007342","fusion of sperm to egg plasma membrane involved in single fertilization","2/183","17/17653",0.0131226161494697,0.222335991656326,0.202736522606266,"FOLR3/NOX5",2 |
| GO:0090185,"BP","GO:0090185","negative regulation of kidney development","2/183","17/17653",0.0131226161494697,0.222335991656326,0.202736522606266,"PAX8/WT1",2 |
| GO:0099068,"BP","GO:0099068","postsynapse assembly","2/183","17/17653",0.0131226161494697,0.222335991656326,0.202736522606266,"WNT7A/CDH2",2 |
| GO:0007389,"BP","GO:0007389","pattern specification process","10/183","424/17653",0.0131835400952617,0.222335991656326,0.202736522606266,"PAX8/TBR1/WNT7A/STC1/SP8/IRX1/IRX2/SOX1/WT1/ARC",10 |
| GO:0090257,"BP","GO:0090257","regulation of muscle system process","7/183","242/17653",0.0132669624650894,0.222335991656326,0.202736522606266,"PKP2/KCNA1/SPX/MYOCD/STC1/ADRA1D/RYR2",7 |
| GO:0098742,"BP","GO:0098742","cell-cell adhesion via plasma-membrane adhesion molecules","7/183","242/17653",0.0132669624650894,0.222335991656326,0.202736522606266,"PCDHB8/CDH12/IL1RAPL1/CDH2/DAB1/PCDH9/PCDHA11",7 |
| GO:0030072,"BP","GO:0030072","peptide hormone secretion","7/183","243/17653",0.0135446832016028,0.225435480684211,0.205562783989718,"PTPRN/HNF4A/GCK/PAX8/VGF/ANO1/VIP",7 |
| GO:0030900,"BP","GO:0030900","forebrain development","9/183","366/17653",0.0143068280785861,0.234286200116348,0.213633290554434,"KCNA1/TBR1/WNT7A/CDH2/DAB1/TH/SOX1/PCDH9/POU3F3",9 |
| GO:0032095,"BP","GO:0032095","regulation of response to food","2/183","18/17653",0.0146632725464097,0.234286200116348,0.213633290554434,"SPX/CCK",2 |
| GO:0071371,"BP","GO:0071371","cellular response to gonadotropin stimulus","2/183","18/17653",0.0146632725464097,0.234286200116348,0.213633290554434,"PAX8/WT1",2 |
| GO:0090330,"BP","GO:0090330","regulation of platelet aggregation","2/183","18/17653",0.0146632725464097,0.234286200116348,0.213633290554434,"ADAMTS18/PDPN",2 |
| GO:0098911,"BP","GO:0098911","regulation of ventricular cardiac muscle cell action potential","2/183","18/17653",0.0146632725464097,0.234286200116348,0.213633290554434,"PKP2/RYR2",2 |
| GO:0046879,"BP","GO:0046879","hormone secretion","8/183","306/17653",0.0146686103363273,0.234286200116348,0.213633290554434,"PTPRN/IL11/HNF4A/GCK/PAX8/VGF/ANO1/VIP",8 |
| GO:0061564,"BP","GO:0061564","axon development","11/183","497/17653",0.0147513533406589,0.234286200116348,0.213633290554434,"NEFH/TBR1/WNT7A/LRRC38/NTNG1/CDH2/NPTX1/DAB1/FLRT2/CCK/L1CAM",11 |
| GO:0045933,"BP","GO:0045933","positive regulation of muscle contraction","3/183","50/17653",0.015034889522743,0.237238841170555,0.216325648977266,"SPX/MYOCD/ADRA1D",3 |
| GO:0001505,"BP","GO:0001505","regulation of neurotransmitter levels","8/183","308/17653",0.0151987763703861,0.238277590838956,0.217272830286334,"NTSR1/SLC22A2/UNC13A/GFAP/SYT16/WNT7A/PTX3/TH",8 |
| GO:0060997,"BP","GO:0060997","dendritic spine morphogenesis","3/183","51/17653",0.0158552075711175,0.245683758722797,0.224026126104175,"WNT7A/CTNND2/ARC",3 |
| GO:0007617,"BP","GO:0007617","mating behavior","2/183","19/17653",0.0162778128207285,0.245683758722797,0.224026126104175,"THRB/TH",2 |
| GO:0010523,"BP","GO:0010523","negative regulation of calcium ion transport into cytosol","2/183","19/17653",0.0162778128207285,0.245683758722797,0.224026126104175,"NTSR1/TRDN",2 |
| GO:0016048,"BP","GO:0016048","detection of temperature stimulus","2/183","19/17653",0.0162778128207285,0.245683758722797,0.224026126104175,"NTSR1/ANO1",2 |
| GO:0023019,"BP","GO:0023019","signal transduction involved in regulation of gene expression","2/183","19/17653",0.0162778128207285,0.245683758722797,0.224026126104175,"HNF4A/FGF5",2 |
| GO:0099054,"BP","GO:0099054","presynapse assembly","2/183","19/17653",0.0162778128207285,0.245683758722797,0.224026126104175,"WNT7A/IL1RAPL1",2 |
| GO:0010676,"BP","GO:0010676","positive regulation of cellular carbohydrate metabolic process","3/183","52/17653",0.0167002769312526,0.249087464629769,0.227129786894374,"NTSR1/GCK/PTH",3 |
| GO:0001764,"BP","GO:0001764","neuron migration","5/183","143/17653",0.0167083361047952,0.249087464629769,0.227129786894374,"DAB1/SOX1/FLRT2/CCK/DNER",5 |
| GO:0035249,"BP","GO:0035249","synaptic transmission, glutamatergic","4/183","95/17653",0.017134007193055,0.25387583828734,0.23149605353903,"GRIA3/UNC13A/CDH2/ADRA1D",4 |
| GO:0098900,"BP","GO:0098900","regulation of action potential","3/183","53/17653",0.01757016111146,0.256787491834918,0.234151037605589,"PKP2/NTSR1/RYR2",3 |
| GO:0055123,"BP","GO:0055123","digestive system development","5/183","145/17653",0.017636893210428,0.256787491834918,0.234151037605589,"NKX2-3/GATA5/MYOCD/DAB1/FOXL1",5 |
| GO:0007188,"BP","GO:0007188","adenylate cyclase-modulating G-protein coupled receptor signaling pathway","6/183","199/17653",0.0177005373675024,0.256787491834918,0.234151037605589,"ADCY2/AKAP12/VIP/PTH/GPR78/ADRA1D",6 |
| GO:0006309,"BP","GO:0006309","apoptotic DNA fragmentation","2/183","20/17653",0.017964557041949,0.256787491834918,0.234151037605589,"DNASE1L3/CIDEA",2 |
| GO:0060231,"BP","GO:0060231","mesenchymal to epithelial transition","2/183","20/17653",0.017964557041949,0.256787491834918,0.234151037605589,"PAX8/WT1",2 |
| GO:0071711,"BP","GO:0071711","basement membrane organization","2/183","20/17653",0.017964557041949,0.256787491834918,0.234151037605589,"LAMC2/FLRT2",2 |
| GO:0002062,"BP","GO:0002062","chondrocyte differentiation","4/183","97/17653",0.0183555055911841,0.259324875503357,0.236464744611032,"WNT5B/HMGA2/PTH/WNT7A",4 |
| GO:0051591,"BP","GO:0051591","response to cAMP","4/183","97/17653",0.0183555055911841,0.259324875503357,0.236464744611032,"PTPRN/VGF/STC1/WT1",4 |
| GO:0045165,"BP","GO:0045165","cell fate commitment","7/183","259/17653",0.0185744594789313,0.259744871884971,0.23684771727003,"WNT5B/GATA5/TBR1/WNT7A/PDPN/SOX1/WT1",7 |
| GO:0014074,"BP","GO:0014074","response to purine-containing compound","5/183","147/17653",0.0185990155176893,0.259744871884971,0.23684771727003,"PTPRN/VGF/STC1/WT1/RYR2",5 |
| GO:0061005,"BP","GO:0061005","cell differentiation involved in kidney development","3/183","55/17653",0.0193845749937121,0.263319230557912,0.240106987361666,"PAX8/WT1/POU3F3",3 |
| GO:0086065,"BP","GO:0086065","cell communication involved in cardiac conduction","3/183","55/17653",0.0193845749937121,0.263319230557912,0.240106987361666,"PKP2/TRDN/RYR2",3 |
| GO:0019933,"BP","GO:0019933","cAMP-mediated signaling","5/183","149/17653",0.0195951449197981,0.263319230557912,0.240106987361666,"ADCY2/VIP/PTH/GPR78/ADRA1D",5 |
| GO:0015874,"BP","GO:0015874","norepinephrine transport","2/183","21/17653",0.0197218518360247,0.263319230557912,0.240106987361666,"SLC22A2/SLC22A1",2 |
| GO:0050951,"BP","GO:0050951","sensory perception of temperature stimulus","2/183","21/17653",0.0197218518360247,0.263319230557912,0.240106987361666,"NTSR1/ANO1",2 |
| GO:0060065,"BP","GO:0060065","uterus development","2/183","21/17653",0.0197218518360247,0.263319230557912,0.240106987361666,"MYOCD/WNT7A",2 |
| GO:0099172,"BP","GO:0099172","presynapse organization","2/183","21/17653",0.0197218518360247,0.263319230557912,0.240106987361666,"WNT7A/IL1RAPL1",2 |
| GO:1902932,"BP","GO:1902932","positive regulation of alcohol biosynthetic process","2/183","21/17653",0.0197218518360247,0.263319230557912,0.240106987361666,"NTSR1/PTH",2 |
| GO:0030817,"BP","GO:0030817","regulation of cAMP biosynthetic process","3/183","57/17653",0.0212987492626859,0.28151510832887,0.256698853381967,"NPFFR2/PTH/ADRA1D",3 |
| GO:0051048,"BP","GO:0051048","negative regulation of secretion","6/183","208/17653",0.0214813436973693,0.28151510832887,0.256698853381967,"IL11/IL13RA2/SPX/NLRP7/IL1RAPL1/CIDEA",6 |
| GO:0046135,"BP","GO:0046135","pyrimidine nucleoside catabolic process","2/183","22/17653",0.0215480700202345,0.28151510832887,0.256698853381967,"NT5E/CDA",2 |
| GO:0061213,"BP","GO:0061213","positive regulation of mesonephros development","2/183","22/17653",0.0215480700202345,0.28151510832887,0.256698853381967,"PAX8/WT1",2 |
| GO:0035821,"BP","GO:0035821","modification of morphology or physiology of other organism","5/183","153/17653",0.0216911108410795,0.281868445688894,0.257021043177441,"APOL1/CRP/HMGA2/PTX3/RNASE7",5 |
| GO:0051051,"BP","GO:0051051","negative regulation of transport","10/183","460/17653",0.0219221421797422,0.281908098388954,0.257057200393654,"IL11/NTSR1/IL13RA2/SPX/STC1/NLRP7/IL1RAPL1/ADRA1D/CIDEA/TRDN",10 |
| GO:0046530,"BP","GO:0046530","photoreceptor cell differentiation","3/183","58/17653",0.0222932970599605,0.281908098388954,0.257057200393654,"RPE65/TH/RP1L1",3 |
| GO:0099175,"BP","GO:0099175","regulation of postsynapse organization","3/183","58/17653",0.0222932970599605,0.281908098388954,0.257057200393654,"CDH2/ARC/GRIN2B",3 |
| GO:0052652,"BP","GO:0052652","cyclic purine nucleotide metabolic process","4/183","103/17653",0.0223391702818039,0.281908098388954,0.257057200393654,"NPFFR2/ADCY2/PTH/ADRA1D",4 |
| GO:0071804,"BP","GO:0071804","cellular potassium ion transport","6/183","210/17653",0.0223902316827441,0.281908098388954,0.257057200393654,"KCNA1/KCNS1/DPP6/KCNJ1/LRRC38/KCNJ12",6 |
| GO:0071805,"BP","GO:0071805","potassium ion transmembrane transport","6/183","210/17653",0.0223902316827441,0.281908098388954,0.257057200393654,"KCNA1/KCNS1/DPP6/KCNJ1/LRRC38/KCNJ12",6 |
| GO:0009190,"BP","GO:0009190","cyclic nucleotide biosynthetic process","4/183","104/17653",0.0230503543557885,0.288723510745186,0.263271817248697,"NPFFR2/ADCY2/PTH/ADRA1D",4 |
| GO:1901016,"BP","GO:1901016","regulation of potassium ion transmembrane transporter activity","3/183","59/17653",0.0233128266479047,0.288868596841121,0.263404113645467,"KCNA1/KCNS1/LRRC38",3 |
| GO:0044062,"BP","GO:0044062","regulation of excretion","2/183","23/17653",0.0234416102427271,0.288868596841121,0.263404113645467,"SPX/STC1",2 |
| GO:0072215,"BP","GO:0072215","regulation of metanephros development","2/183","23/17653",0.0234416102427271,0.288868596841121,0.263404113645467,"PAX8/WT1",2 |
| GO:0007218,"BP","GO:0007218","neuropeptide signaling pathway","4/183","105/17653",0.0237751931556478,0.288868596841121,0.263404113645467,"NPFFR2/NTSR1/SORCS1/ECEL1",4 |
| GO:0045446,"BP","GO:0045446","endothelial cell differentiation","4/183","105/17653",0.0237751931556478,0.288868596841121,0.263404113645467,"WNT7A/XDH/STC1/PDPN",4 |
| GO:0046058,"BP","GO:0046058","cAMP metabolic process","4/183","105/17653",0.0237751931556478,0.288868596841121,0.263404113645467,"NPFFR2/ADCY2/PTH/ADRA1D",4 |
| GO:0048645,"BP","GO:0048645","animal organ formation","3/183","60/17653",0.0243573326561562,0.29339060248213,0.267527493274803,"PAX8/TBR1/WT1",3 |
| GO:0007156,"BP","GO:0007156","homophilic cell adhesion via plasma membrane adhesion molecules","5/183","158/17653",0.0245095853102355,0.29339060248213,0.267527493274803,"PCDHB8/CDH12/CDH2/PCDH9/PCDHA11",5 |
| GO:0015718,"BP","GO:0015718","monocarboxylic acid transport","5/183","158/17653",0.0245095853102355,0.29339060248213,0.267527493274803,"NTSR1/SLCO1B3/SLC27A6/SLC6A11/SPX",5 |
| GO:0032098,"BP","GO:0032098","regulation of appetite","2/183","24/17653",0.0254008966266602,0.296750859628771,0.270591535420647,"SPX/CCK",2 |
| GO:0032104,"BP","GO:0032104","regulation of response to extracellular stimulus","2/183","24/17653",0.0254008966266602,0.296750859628771,0.270591535420647,"SPX/CCK",2 |
| GO:0032107,"BP","GO:0032107","regulation of response to nutrient levels","2/183","24/17653",0.0254008966266602,0.296750859628771,0.270591535420647,"SPX/CCK",2 |
| GO:0097186,"BP","GO:0097186","amelogenesis","2/183","24/17653",0.0254008966266602,0.296750859628771,0.270591535420647,"MMP20/KLK4",2 |
| GO:1901018,"BP","GO:1901018","positive regulation of potassium ion transmembrane transporter activity","2/183","24/17653",0.0254008966266602,0.296750859628771,0.270591535420647,"KCNA1/LRRC38",2 |
| GO:0050890,"BP","GO:0050890","cognition","7/183","277/17653",0.0256594164707531,0.29738469871808,0.271169500022334,"NTSR1/CALB1/TBR1/AFF2/TH/ARC/GRIN2B",7 |
| GO:0019935,"BP","GO:0019935","cyclic-nucleotide-mediated signaling","5/183","160/17653",0.0256999122348958,0.29738469871808,0.271169500022334,"ADCY2/VIP/PTH/GPR78/ADRA1D",5 |
| GO:0048593,"BP","GO:0048593","camera-type eye morphogenesis","4/183","108/17653",0.0260321521973555,0.297796721464555,0.271545201941906,"CALB1/RPE65/TH/SOX1",4 |
| GO:0051928,"BP","GO:0051928","positive regulation of calcium ion transport","4/183","108/17653",0.0260321521973555,0.297796721464555,0.271545201941906,"NTSR1/STC1/TRDN/RYR2",4 |
| GO:0033500,"BP","GO:0033500","carbohydrate homeostasis","6/183","218/17653",0.0262847048218115,0.297796721464555,0.271545201941906,"PTPRN/HNF4A/GCK/VGF/ANO1/TH",6 |
| GO:0042593,"BP","GO:0042593","glucose homeostasis","6/183","218/17653",0.0262847048218115,0.297796721464555,0.271545201941906,"PTPRN/HNF4A/GCK/VGF/ANO1/TH",6 |
| GO:0019932,"BP","GO:0019932","second-messenger-mediated signaling","8/183","342/17653",0.0264733818724453,0.297796721464555,0.271545201941906,"ADCY2/VIP/PTH/GPR78/ADRA1D/TRDN/RYR2/GRIN2B",8 |
| GO:0015909,"BP","GO:0015909","long-chain fatty acid transport","3/183","62/17653",0.026521208663761,0.297796721464555,0.271545201941906,"NTSR1/SLC27A6/SPX",3 |
| GO:0072507,"BP","GO:0072507","divalent inorganic cation homeostasis","10/183","475/17653",0.0265933697768759,0.297796721464555,0.271545201941906,"NTSR1/CALB1/KCNA1/PLCZ1/PTH/STC1/ADRA1D/TRDN/RYR2/GRIN2B",10 |
| GO:0031012,"CC","GO:0031012","extracellular matrix","17/192","479/18698",9.57668693563308e-06,0.00218348462132434,0.00187501449476606,"LAMA3/LAMC2/WNT5B/RTBDN/MMP20/ADAMTS18/TINAGL1/LEFTY2/WNT7A/LRRC38/COL22A1/CDH2/FLRT2/MFAP5/SMOC1/L1CAM/MMP28",17 |
| GO:0005578,"CC","GO:0005578","proteinaceous extracellular matrix","13/192","375/18698",0.000139312048051891,0.0117545602724631,0.0100939437242758,"LAMA3/LAMC2/WNT5B/RTBDN/MMP20/ADAMTS18/WNT7A/LRRC38/COL22A1/FLRT2/MFAP5/SMOC1/MMP28",13 |
| GO:0098793,"CC","GO:0098793","presynapse","13/192","379/18698",0.000154665266742936,0.0117545602724631,0.0100939437242758,"PTPRN/NTSR1/CALB1/KCNA1/SLC22A2/UNC13A/WNT7A/CDH2/ADRA1D/TH/KCTD8/CCK/SLC6A17",13 |
| GO:0043679,"CC","GO:0043679","axon terminus","7/192","123/18698",0.000281024394379716,0.0160183904796438,0.0137554045670072,"PTPRN/NTSR1/CALB1/KCNA1/UNC13A/TH/CCK",7 |
| GO:0034703,"CC","GO:0034703","cation channel complex","9/192","217/18698",0.000418581273235036,0.017714233441205,0.0152116686060209,"KCNA1/KCNS1/GRIA3/DPP6/KCNJ1/LRRC38/TRDN/RYR2/GRIN2B",9 |
| GO:0150034,"CC","GO:0150034","distal axon","10/192","268/18698",0.000466164037926448,0.017714233441205,0.0152116686060209,"STMN4/PTPRN/NTSR1/CALB1/KCNA1/UNC13A/TH/PCDH9/CCK/L1CAM",10 |
| GO:0044306,"CC","GO:0044306","neuron projection terminus","7/192","139/18698",0.000588438090183811,0.0191662692231298,0.0164585691389758,"PTPRN/NTSR1/CALB1/KCNA1/UNC13A/TH/CCK",7 |
| GO:0043195,"CC","GO:0043195","terminal bouton","5/192","71/18698",0.000815117063069268,0.0232308362974742,0.0199489175961689,"NTSR1/CALB1/UNC13A/TH/CCK",5 |
| GO:0034702,"CC","GO:0034702","ion channel complex","10/192","296/18698",0.00100064376581226,0.0253496420672438,0.0217683906948631,"KCNA1/KCNS1/GRIA3/DPP6/ANO1/KCNJ1/LRRC38/TRDN/RYR2/GRIN2B",10 |
| GO:0043204,"CC","GO:0043204","perikaryon","6/192","118/18698",0.00137949015996858,0.0314523756472836,0.0270089652372795,"PTPRN/NTSR1/KCNA1/CTNND2/TH/CCK",6 |
| GO:1902495,"CC","GO:1902495","transmembrane transporter complex","10/192","321/18698",0.00182976682839067,0.0379260760793703,0.0325680985723124,"KCNA1/KCNS1/GRIA3/DPP6/ANO1/KCNJ1/LRRC38/TRDN/RYR2/GRIN2B",10 |
| GO:1990351,"CC","GO:1990351","transporter complex","10/192","329/18698",0.00218959510411327,0.0416023069781522,0.0357249727513218,"KCNA1/KCNS1/GRIA3/DPP6/ANO1/KCNJ1/LRRC38/TRDN/RYR2/GRIN2B",10 |
| GO:0043025,"CC","GO:0043025","neuronal cell body","12/192","449/18698",0.00240002876343717,0.0420928121587443,0.0361461821861793,"PTPRN/NTSR1/CALB1/KCNA1/BRINP3/CTNND2/DAB1/TH/CCK/DNER/ARC/L1CAM",12 |
| GO:0008076,"CC","GO:0008076","voltage-gated potassium channel complex","5/192","92/18698",0.00258978538011329,0.042176504761845,0.0362180511805317,"KCNA1/KCNS1/DPP6/KCNJ1/LRRC38",5 |
| GO:0034705,"CC","GO:0034705","potassium channel complex","5/192","99/18698",0.0035562622577667,0.0516223328573098,0.0443294271073852,"KCNA1/KCNS1/DPP6/KCNJ1/LRRC38",5 |
| GO:0033267,"CC","GO:0033267","axon part","10/192","353/18698",0.00362261984963578,0.0516223328573098,0.0443294271073852,"STMN4/PTPRN/NTSR1/CALB1/KCNA1/UNC13A/TH/PCDH9/CCK/L1CAM",10 |
| GO:0099634,"CC","GO:0099634","postsynaptic specialization membrane","3/192","34/18698",0.00504694743728959,0.0666283722014134,0.0572154996743439,"GRIA3/CDH2/GRIN2B",3 |
| GO:0044291,"CC","GO:0044291","cell-cell contact zone","4/192","68/18698",0.00526013464748001,0.0666283722014134,0.0572154996743439,"PKP2/CDH2/PCDH9/AJAP1",4 |
| GO:0098794,"CC","GO:0098794","postsynapse","11/192","450/18698",0.00695142401917023,0.0834170882300427,0.0716324026352167,"NEFH/NTSR1/CALB1/GRIA3/IL1RAPL1/FRMPD4/CDH2/DAB1/KCTD8/ARC/GRIN2B",11 |
| GO:0042734,"CC","GO:0042734","presynaptic membrane","4/192","79/18698",0.00889688563347772,0.101424496221646,0.0870958277803609,"KCNA1/UNC13A/CDH2/KCTD8",4 |
| GO:0098984,"CC","GO:0098984","neuron to neuron synapse","7/192","235/18698",0.0109260516367129,0.118625703484311,0.101866947590406,"NEFH/NTSR1/GRIA3/CDH2/DAB1/ARC/GRIN2B",7 |
| GO:0005788,"CC","GO:0005788","endoplasmic reticulum lumen","8/192","297/18698",0.011847772122588,0.12278600199773,0.105439503100544,"APOL1/WNT5B/VGF/ERP27/WNT7A/COL22A1/CDH2/TRDN",8 |
| GO:0046658,"CC","GO:0046658","anchored component of plasma membrane","3/192","48/18698",0.0131386379271604,0.130243889017069,0.111843782812441,"FOLR3/RTBDN/NTNG1",3 |
| GO:0031362,"CC","GO:0031362","anchored component of external side of plasma membrane","2/192","18/18698",0.0144049517438109,0.136847041566204,0.1175140800153,"FOLR3/RTBDN",2 |
| GO:0097060,"CC","GO:0097060","synaptic membrane","8/192","317/18698",0.0169237761028735,0.154344838058207,0.132539888637241,"KCNA1/GRIA3/UNC13A/IL1RAPL1/CDH2/KCTD8/ARC/GRIN2B",8 |
| GO:1904724,"CC","GO:1904724","tertiary granule lumen","3/192","55/18698",0.0189187809425841,0.163635657497039,0.140518154637346,"FOLR3/CDA/PTX3",3 |
| GO:0005605,"CC","GO:0005605","basal lamina","2/192","21/18698",0.0193779068088598,0.163635657497039,0.140518154637346,"LAMA3/LAMC2",2 |
| GO:0016323,"CC","GO:0016323","basolateral plasma membrane","6/192","210/18698",0.0215285336571928,0.175303774065712,0.150537866926235,"SLCO1B3/SLC14A1/PDPN/CDH2/SLC22A1/AJAP1",6 |
| GO:0031233,"CC","GO:0031233","intrinsic component of external side of plasma membrane","2/192","23/18698",0.0230355502637261,0.181107084832054,0.155521319384866,"FOLR3/RTBDN",2 |
| GO:0031225,"CC","GO:0031225","anchored component of membrane","5/192","159/18698",0.0242504725508149,0.184303591386194,0.158266241910582,"FOLR3/RTBDN/NT5E/NTNG1/ALPP",5 |
| GO:0016529,"CC","GO:0016529","sarcoplasmic reticulum","3/192","62/18698",0.025895538472211,0.190457508763358,0.163550769298175,"XDH/TRDN/RYR2",3 |
| GO:0014069,"CC","GO:0014069","postsynaptic density","6/192","227/18698",0.0300110102904235,0.213828448319267,0.18361999717167,"NEFH/GRIA3/CDH2/DAB1/ARC/GRIN2B",6 |
| GO:0099572,"CC","GO:0099572","postsynaptic specialization","6/192","229/18698",0.0311343274218171,0.215109898550736,0.184720411497862,"NEFH/GRIA3/CDH2/DAB1/ARC/GRIN2B",6 |
| GO:0032279,"CC","GO:0032279","asymmetric synapse","6/192","232/18698",0.0328703276711842,0.219732504116493,0.188689961983692,"NEFH/GRIA3/CDH2/DAB1/ARC/GRIN2B",6 |
| GO:0044420,"CC","GO:0044420","extracellular matrix component","4/192","119/18698",0.0343792648707334,0.219732504116493,0.188689961983692,"LAMA3/LAMC2/MFAP5/SMOC1",4 |
| GO:0016342,"CC","GO:0016342","catenin complex","2/192","29/18698",0.0355169226337056,0.219732504116493,0.188689961983692,"CDH12/CDH2",2 |
| GO:0016459,"CC","GO:0016459","myosin complex","3/192","71/18698",0.0366220840194154,0.219732504116493,0.188689961983692,"MYH16/MYO16/MYH15",3 |
| GO:0016528,"CC","GO:0016528","sarcoplasm","3/192","71/18698",0.0366220840194154,0.219732504116493,0.188689961983692,"XDH/TRDN/RYR2",3 |
| GO:0045211,"CC","GO:0045211","postsynaptic membrane","6/192","244/18698",0.0404385183029869,0.23640979930977,0.203011185002849,"GRIA3/IL1RAPL1/CDH2/KCTD8/ARC/GRIN2B",6 |
| GO:0098839,"CC","GO:0098839","postsynaptic density membrane","2/192","32/18698",0.0425344048183227,0.241628638505796,0.207492736667027,"GRIA3/GRIN2B",2 |
| GO:0030133,"CC","GO:0030133","transport vesicle","8/192","381/18698",0.0434507639418318,0.241628638505796,0.207492736667027,"PTPRN/VGF/UNC13A/SPX/NPTX1/ADRA1D/TH/SLC6A17",8 |
| GO:0005790,"CC","GO:0005790","smooth endoplasmic reticulum","2/192","33/18698",0.0449794454564588,0.244174132477919,0.209678617917327,"TH/RYR2",2 |
| GO:0048786,"CC","GO:0048786","presynaptic active zone","2/192","35/18698",0.0500204604112932,0.265224766831973,0.227755339938814,"UNC13A/CDH2",2 |
| GO:0098562,"CC","GO:0098562","cytoplasmic side of membrane","5/192","199/18698",0.0547241639909097,0.283570667952896,0.243509437854287,"NTSR1/GFAP/TH/RGS7/AJAP1",5 |
| GO:0048018,"MF","GO:0048018","receptor ligand activity","15/179","472/17548",0.000102815537117492,0.0432853411264642,0.0378794084117076,"IL11/CSF3/IL1A/VGF/SPX/FGF5/LEFTY2/VIP/PTH/WNT7A/STC1/IL24/FLRT2/CCK/VSTM1",15 |
| GO:0005184,"MF","GO:0005184","neuropeptide hormone activity","4/179","30/17548",0.000233180017513559,0.0460272408550076,0.0402788706069575,"VGF/SPX/VIP/CCK",4 |
| GO:0005261,"MF","GO:0005261","cation channel activity","11/179","316/17548",0.000411287747015517,0.0460272408550076,0.0402788706069575,"KCNA1/KCNS1/GRIA3/ANO1/KCNJ1/LRRC38/IL1RAPL1/KCNJ12/RYR2/NOX5/GRIN2B",11 |
| GO:0022838,"MF","GO:0022838","substrate-specific channel activity","13/179","437/17548",0.000560552888456863,0.0460272408550076,0.0402788706069575,"APOL1/KCNA1/KCNS1/GRIA3/ANO1/SLC14A1/KCNJ1/LRRC38/IL1RAPL1/KCNJ12/RYR2/NOX5/GRIN2B",13 |
| GO:0022843,"MF","GO:0022843","voltage-gated cation channel activity","7/179","143/17548",0.000666659123466719,0.0460272408550076,0.0402788706069575,"KCNA1/KCNS1/KCNJ1/LRRC38/IL1RAPL1/KCNJ12/GRIN2B",7 |
| GO:0015267,"MF","GO:0015267","channel activity","13/179","463/17548",0.000954946512486915,0.0460272408550076,0.0402788706069575,"APOL1/KCNA1/KCNS1/GRIA3/ANO1/SLC14A1/KCNJ1/LRRC38/IL1RAPL1/KCNJ12/RYR2/NOX5/GRIN2B",13 |
| GO:0022803,"MF","GO:0022803","passive transmembrane transporter activity","13/179","464/17548",0.000973868020464452,0.0460272408550076,0.0402788706069575,"APOL1/KCNA1/KCNS1/GRIA3/ANO1/SLC14A1/KCNJ1/LRRC38/IL1RAPL1/KCNJ12/RYR2/NOX5/GRIN2B",13 |
| GO:0005244,"MF","GO:0005244","voltage-gated ion channel activity","8/179","198/17548",0.000983955267684247,0.0460272408550076,0.0402788706069575,"KCNA1/KCNS1/ANO1/KCNJ1/LRRC38/IL1RAPL1/KCNJ12/GRIN2B",8 |
| GO:0022832,"MF","GO:0022832","voltage-gated channel activity","8/179","198/17548",0.000983955267684247,0.0460272408550076,0.0402788706069575,"KCNA1/KCNS1/ANO1/KCNJ1/LRRC38/IL1RAPL1/KCNJ12/GRIN2B",8 |
| GO:0005216,"MF","GO:0005216","ion channel activity","12/179","424/17548",0.00139629942915743,0.0578602231837427,0.0506340245388422,"APOL1/KCNA1/KCNS1/GRIA3/ANO1/KCNJ1/LRRC38/IL1RAPL1/KCNJ12/RYR2/NOX5/GRIN2B",12 |
| GO:0005179,"MF","GO:0005179","hormone activity","6/179","121/17548",0.00151178730408829,0.0578602231837427,0.0506340245388422,"VGF/SPX/VIP/PTH/STC1/CCK",6 |
| GO:0005326,"MF","GO:0005326","neurotransmitter transporter activity","4/179","54/17548",0.00222499255679182,0.0682831070933075,0.0597551881051572,"SLC22A2/SLC6A11/SLC22A1/SLC6A17",4 |
| GO:0022839,"MF","GO:0022839","ion gated channel activity","10/179","335/17548",0.00236089595933983,0.0682831070933075,0.0597551881051572,"KCNA1/KCNS1/GRIA3/ANO1/KCNJ1/LRRC38/IL1RAPL1/KCNJ12/RYR2/GRIN2B",10 |
| GO:0008514,"MF","GO:0008514","organic anion transmembrane transporter activity","8/179","229/17548",0.00246359012774837,0.0682831070933075,0.0597551881051572,"SLCO1B3/SLC22A2/SLC27A6/RTBDN/SLC6A11/SLC13A5/SLC22A1/SLC6A17",8 |
| GO:0022836,"MF","GO:0022836","gated channel activity","10/179","338/17548",0.00251778602617591,0.0682831070933075,0.0597551881051572,"KCNA1/KCNS1/GRIA3/ANO1/KCNJ1/LRRC38/IL1RAPL1/KCNJ12/RYR2/GRIN2B",10 |
| GO:0008509,"MF","GO:0008509","anion transmembrane transporter activity","10/179","342/17548",0.00273988735781728,0.0682831070933075,0.0597551881051572,"APOL1/SLCO1B3/SLC22A2/SLC27A6/ANO1/RTBDN/SLC6A11/SLC13A5/SLC22A1/SLC6A17",10 |
| GO:0005249,"MF","GO:0005249","voltage-gated potassium channel activity","5/179","94/17548",0.00275727510828083,0.0682831070933075,0.0597551881051572,"KCNA1/KCNS1/KCNJ1/LRRC38/KCNJ12",5 |
| GO:0015651,"MF","GO:0015651","quaternary ammonium group transmembrane transporter activity","2/179","10/17548",0.00441241998909947,0.100767106050482,0.0881822405742435,"SLC22A2/SLC22A1",2 |
| GO:0015347,"MF","GO:0015347","sodium-independent organic anion transmembrane transporter activity","3/179","33/17548",0.00454768412104313,0.100767106050482,0.0881822405742435,"SLCO1B3/SLC22A2/SLC22A1",3 |
| GO:0099094,"MF","GO:0099094","ligand-gated cation channel activity","5/179","109/17548",0.00519473687825691,0.107393565203461,0.0939811171926777,"GRIA3/KCNJ1/KCNJ12/RYR2/GRIN2B",5 |
| GO:0045294,"MF","GO:0045294","alpha-catenin binding","2/179","11/17548",0.00535692367998263,0.107393565203461,0.0939811171926777,"PKP2/CDH2",2 |
| GO:0070405,"MF","GO:0070405","ammonium ion binding","4/179","75/17548",0.00724966298191919,0.126233180440378,0.110467841365501,"RPE65/CRP/ADRA1D/TH",4 |
| GO:0046873,"MF","GO:0046873","metal ion transmembrane transporter activity","11/179","457/17548",0.00735006943722905,0.126233180440378,0.110467841365501,"KCNA1/KCNS1/SLC6A11/SLC13A5/KCNJ1/LRRC38/IL1RAPL1/KCNJ12/SLC6A17/RYR2/GRIN2B",11 |
| GO:0005125,"MF","GO:0005125","cytokine activity","7/179","220/17548",0.00747796458432389,0.126233180440378,0.110467841365501,"IL11/CSF3/IL1A/LEFTY2/WNT7A/IL24/VSTM1",7 |
| GO:0008179,"MF","GO:0008179","adenylate cyclase binding","2/179","13/17548",0.007496032092659,0.126233180440378,0.110467841365501,"ADCY2/AKAP12",2 |
| GO:0035240,"MF","GO:0035240","dopamine binding","2/179","14/17548",0.00868708076875886,0.140663884755672,0.123096286196988,"ADRA1D/TH",2 |
| GO:0001846,"MF","GO:0001846","opsonin binding","2/179","15/17548",0.00995680046146538,0.149707606938462,0.131010532387702,"CRP/PTX3",2 |
| GO:0070742,"MF","GO:0070742","C2H2 zinc finger domain binding","2/179","15/17548",0.00995680046146538,0.149707606938462,0.131010532387702,"HMGA2/WT1",2 |
| GO:0005267,"MF","GO:0005267","potassium channel activity","5/179","129/17548",0.0104089185051915,0.151108782437436,0.132236714222034,"KCNA1/KCNS1/KCNJ1/LRRC38/KCNJ12",5 |
| GO:1901618,"MF","GO:1901618","organic hydroxy compound transmembrane transporter activity","3/179","46/17548",0.0114890848914871,0.159827849124811,0.139866851340627,"SLCO1B3/SLC22A2/SLC22A1",3 |
| GO:0070851,"MF","GO:0070851","growth factor receptor binding","5/179","133/17548",0.0117687964913756,0.159827849124811,0.139866851340627,"IL11/CSF3/IL1A/FGF5/FLRT2",5 |
| GO:0015459,"MF","GO:0015459","potassium channel regulator activity","3/179","49/17548",0.013638516064831,0.170830529100797,0.149495399888183,"KCNS1/DPP6/LRRC38",3 |
| GO:0043177,"MF","GO:0043177","organic acid binding","6/179","191/17548",0.0137126594498503,0.170830529100797,0.149495399888183,"HNF4A/FOLR3/TH/CYP27C1/RYR2/GRIN2B",6 |
| GO:0008301,"MF","GO:0008301","DNA binding, bending","2/179","18/17548",0.014221012426253,0.170830529100797,0.149495399888183,"HMGA2/FOXL1",2 |
| GO:0008188,"MF","GO:0008188","neuropeptide receptor activity","3/179","51/17548",0.0151899438672702,0.170830529100797,0.149495399888183,"NPFFR2/NTSR1/SORCS1",3 |
| GO:0004970,"MF","GO:0004970","ionotropic glutamate receptor activity","2/179","19/17548",0.0157885734029401,0.170830529100797,0.149495399888183,"GRIA3/GRIN2B",2 |
| GO:0005234,"MF","GO:0005234","extracellularly glutamate-gated ion channel activity","2/179","19/17548",0.0157885734029401,0.170830529100797,0.149495399888183,"GRIA3/GRIN2B",2 |
| GO:0042165,"MF","GO:0042165","neurotransmitter binding","3/179","53/17548",0.0168369691094894,0.170830529100797,0.149495399888183,"SLC6A11/CRP/GRIN2B",3 |
| GO:0015276,"MF","GO:0015276","ligand-gated ion channel activity","5/179","146/17548",0.0170117143019391,0.170830529100797,0.149495399888183,"GRIA3/KCNJ1/KCNJ12/RYR2/GRIN2B",5 |
| GO:0022834,"MF","GO:0022834","ligand-gated channel activity","5/179","146/17548",0.0170117143019391,0.170830529100797,0.149495399888183,"GRIA3/KCNJ1/KCNJ12/RYR2/GRIN2B",5 |
| GO:0015238,"MF","GO:0015238","drug transmembrane transporter activity","4/179","99/17548",0.0186185890778979,0.170830529100797,0.149495399888183,"SLC22A2/RTBDN/SLC13A5/SLC22A1",4 |
| GO:0001848,"MF","GO:0001848","complement binding","2/179","21/17548",0.019133249711966,0.170830529100797,0.149495399888183,"CRP/PTX3",2 |
| GO:0001871,"MF","GO:0001871","pattern binding","2/179","21/17548",0.019133249711966,0.170830529100797,0.149495399888183,"TINAGL1/PTX3",2 |
| GO:0005242,"MF","GO:0005242","inward rectifier potassium channel activity","2/179","21/17548",0.019133249711966,0.170830529100797,0.149495399888183,"KCNJ1/KCNJ12",2 |
| GO:0030247,"MF","GO:0030247","polysaccharide binding","2/179","21/17548",0.019133249711966,0.170830529100797,0.149495399888183,"TINAGL1/PTX3",2 |
| GO:1901338,"MF","GO:1901338","catecholamine binding","2/179","21/17548",0.019133249711966,0.170830529100797,0.149495399888183,"ADRA1D/TH",2 |
| GO:0008028,"MF","GO:0008028","monocarboxylic acid transmembrane transporter activity","3/179","56/17548",0.0194878289066182,0.170830529100797,0.149495399888183,"SLCO1B3/SLC27A6/SLC6A11",3 |
| GO:0015077,"MF","GO:0015077","monovalent inorganic cation transmembrane transporter activity","9/179","393/17548",0.0197016052722429,0.170830529100797,0.149495399888183,"KCNA1/KCNS1/SLC6A11/SLC13A5/KCNJ1/LRRC38/KCNJ12/SLC6A17/NOX5",9 |
| GO:0003705,"MF","GO:0003705","transcription factor activity, RNA polymerase II distal enhancer sequence-specific binding","4/179","101/17548",0.0198828881851284,0.170830529100797,0.149495399888183,"HNF4A/VGLL1/GATA5/PTH",4 |
| GO:0005342,"MF","GO:0005342","organic acid transmembrane transporter activity","5/179","157/17548",0.0225042585490744,0.185770448022752,0.162569463202808,"SLCO1B3/SLC27A6/SLC6A11/SLC13A5/SLC6A17",5 |
| GO:0046943,"MF","GO:0046943","carboxylic acid transmembrane transporter activity","5/179","157/17548",0.0225042585490744,0.185770448022752,0.162569463202808,"SLCO1B3/SLC27A6/SLC6A11/SLC13A5/SLC6A17",5 |
| GO:0001077,"MF","GO:0001077","transcriptional activator activity, RNA polymerase II proximal promoter sequence-specific DNA binding","7/179","279/17548",0.0245818246458666,0.199018234152112,0.174162725223751,"HNF4A/TOX2/PAX8/MYOCD/HMGA2/SOX1/WT1",7 |
| GO:0008083,"MF","GO:0008083","growth factor activity","5/179","163/17548",0.025934408008934,0.206007278712476,0.180278903736383,"IL11/CSF3/VGF/FGF5/LEFTY2",5 |
| GO:0015079,"MF","GO:0015079","potassium ion transmembrane transporter activity","5/179","166/17548",0.0277683084993295,0.211572547222265,0.185149122459789,"KCNA1/KCNS1/KCNJ1/LRRC38/KCNJ12",5 |
| GO:0004222,"MF","GO:0004222","metalloendopeptidase activity","4/179","112/17548",0.0277743944495839,0.211572547222265,0.185149122459789,"MMP20/ADAMTS18/ECEL1/MMP28",4 |
| GO:0005104,"MF","GO:0005104","fibroblast growth factor receptor binding","2/179","26/17548",0.0286452142319931,0.211572547222265,0.185149122459789,"FGF5/FLRT2",2 |
| GO:0015101,"MF","GO:0015101","organic cation transmembrane transporter activity","2/179","26/17548",0.0286452142319931,0.211572547222265,0.185149122459789,"SLC22A2/SLC22A1",2 |
| GO:0005328,"MF","GO:0005328","neurotransmitter:sodium symporter activity","2/179","27/17548",0.030732862799363,0.217985803584076,0.190761423313981,"SLC6A11/SLC6A17",2 |
| GO:0008066,"MF","GO:0008066","glutamate receptor activity","2/179","27/17548",0.030732862799363,0.217985803584076,0.190761423313981,"GRIA3/GRIN2B",2 |
| GO:0005546,"MF","GO:0005546","phosphatidylinositol-4,5-bisphosphate binding","3/179","67/17548",0.0310668603682768,0.217985803584076,0.190761423313981,"PLCZ1/KCNJ1/FRMPD4",3 |
| GO:0044325,"MF","GO:0044325","ion channel binding","4/179","119/17548",0.0336421176268771,0.232185762637955,0.203187940800811,"PKP2/LRRC38/TRDN/RYR2",4 |
| GO:0005251,"MF","GO:0005251","delayed rectifier potassium channel activity","2/179","29/17548",0.0350819455953625,0.234436493581708,0.205157576581067,"KCNA1/KCNS1",2 |
| GO:0099604,"MF","GO:0099604","ligand-gated calcium channel activity","2/179","29/17548",0.0350819455953625,0.234436493581708,0.205157576581067,"RYR2/GRIN2B",2 |
| GO:0005343,"MF","GO:0005343","organic acid:sodium symporter activity","2/179","30/17548",0.0373406136447969,0.24563122413218,0.214954190389456,"SLC6A11/SLC13A5",2 |
| GO:0015291,"MF","GO:0015291","secondary active transmembrane transporter activity","6/179","242/17548",0.0379912256972328,0.246066246439,0.215334882494437,"SLCO1B3/SLC22A2/SLC6A11/SLC13A5/SLC22A1/SLC6A17",6 |
| GO:0008519,"MF","GO:0008519","ammonium transmembrane transporter activity","2/179","31/17548",0.0396535585159979,0.252941638412653,0.221351602561392,"SLC22A2/SLC22A1",2 |
| GO:0008237,"MF","GO:0008237","metallopeptidase activity","5/179","185/17548",0.0412873077191166,0.259432187309673,0.227031542838819,"TRHDE/MMP20/ADAMTS18/ECEL1/MMP28",5 |
| GO:0015370,"MF","GO:0015370","solute:sodium symporter activity","3/179","76/17548",0.0426919135839423,0.26431317086529,0.2313029373743,"SLC6A11/SLC13A5/SLC6A17",3 |
| GO:0005452,"MF","GO:0005452","inorganic anion exchanger activity","2/179","33/17548",0.0444369848064489,0.26801117205066,0.234539092930944,"SLC22A2/SLC22A1",2 |
| GO:0031406,"MF","GO:0031406","carboxylic acid binding","5/179","189/17548",0.0445624276568793,0.26801117205066,0.234539092930944,"HNF4A/FOLR3/TH/CYP27C1/GRIN2B",5 |
| GO:0016247,"MF","GO:0016247","channel regulator activity","4/179","134/17548",0.048475647896606,0.287440109358748,0.251541538381202,"PKP2/KCNS1/DPP6/LRRC38",4 |

Table S4. The information of KEGG analysis based on the identified genes

| ,"ID","Description","GeneRatio","BgRatio","pvalue","p.adjust","qvalue","geneID","Count" |
| --- |
| hsa05031,"hsa05031","Amphetamine addiction","4/76","69/8035",0.00401563155512565,0.37673361876929,0.372527661302966,"2892/7054/23237/2904",4 |
| hsa04080,"hsa04080","Neuroactive ligand-receptor interaction","9/76","340/8035",0.00456646810629443,0.37673361876929,0.372527661302966,"10886/4923/2892/7432/7068/5741/146/885/2904",9 |
| hsa04911,"hsa04911","Insulin secretion","4/76","86/8035",0.00874511012277686,0.398036781504545,0.393592989685515,"108/2645/885/6262",4 |
| hsa04020,"hsa04020","Calcium signaling pathway","6/76","193/8035",0.00964937652132231,0.398036781504545,0.393592989685515,"108/4923/89869/146/10345/6262",6 |
| hsa04713,"hsa04713","Circadian entrainment","4/76","97/8035",0.0132029877339851,0.435698595221508,0.430834336582672,"108/2892/6262/2904",4 |
| hsa04630,"hsa04630","JAK-STAT signaling pathway","5/76","162/8035",0.0183448098019459,0.504482269553511,0.498850091105546,"3589/1440/3598/2670/11009",5 |
| hsa00790,"hsa00790","Folate biosynthesis","2/76","26/8035",0.0247823012540401,0.511134963364576,0.505428512417922,"250/7054",2 |
| hsa04950,"hsa04950","Maturity onset diabetes of the young","2/76","26/8035",0.0247823012540401,0.511134963364576,0.505428512417922,"3172/2645",2 |
| hsa04976,"hsa04976","Bile secretion","3/76","72/8035",0.0304460927344219,0.558178366797734,0.551946710390104,"108/28234/6580",3 |
| hsa05412,"hsa05412","Arrhythmogenic right ventricular cardiomyopathy (ARVC)","3/76","77/8035",0.0361217456644374,0.596008803463217,0.589354797682926,"5318/1000/6262",3 |
| hsa04024,"hsa04024","cAMP signaling pathway","5/76","216/8035",0.053246866505536,0.617674928886782,0.610779036538763,"108/2892/7432/6262/2904",5 |
| hsa05033,"hsa05033","Nicotine addiction","2/76","40/8035",0.0546635364507017,0.617674928886782,0.610779036538763,"2892/2904",2 |
| hsa05323,"hsa05323","Rheumatoid arthritis","3/76","93/8035",0.0576399552968618,0.617674928886782,0.610779036538763,"3589/3552/5741",3 |
| hsa04060,"hsa04060","Cytokine-cytokine receptor interaction","6/76","294/8035",0.0590058941381568,0.617674928886782,0.610779036538763,"3589/1440/3552/3598/11009/8793",6 |
| hsa04940,"hsa04940","Type I diabetes mellitus","2/76","43/8035",0.0621563523706163,0.617674928886782,0.610779036538763,"5798/3552",2 |
| hsa05017,"hsa05017","Spinocerebellar ataxia","3/76","98/8035",0.0653697029637351,0.617674928886782,0.610779036538763,"2892/1600/2904",3 |
| hsa04640,"hsa04640","Hematopoietic cell lineage","3/76","99/8035",0.0669705157672546,0.617674928886782,0.610779036538763,"3589/1440/3552",3 |
| hsa04916,"hsa04916","Melanogenesis","3/76","101/8035",0.0702259367582558,0.617674928886782,0.610779036538763,"108/81029/7476",3 |
| hsa04972,"hsa04972","Pancreatic secretion","3/76","102/8035",0.0718802678730333,0.617674928886782,0.610779036538763,"108/885/6262",3 |
| hsa05030,"hsa05030","Cocaine addiction","2/76","49/8035",0.0780993244078786,0.617674928886782,0.610779036538763,"7054/2904",2 |
| hsa04922,"hsa04922","Glucagon signaling pathway","3/76","106/8035",0.0786717355049005,0.617674928886782,0.610779036538763,"108/2645/3948",3 |
| hsa04961,"hsa04961","Endocrine and other factor-regulated calcium reabsorption","2/76","53/8035",0.0893627962676145,0.617674928886782,0.610779036538763,"793/5741",2 |
| hsa04724,"hsa04724","Glutamatergic synapse","3/76","114/8035",0.0930594905705402,0.617674928886782,0.610779036538763,"108/2892/2904",3 |
| hsa05202,"hsa05202","Transcriptional misregulation in cancer","4/76","186/8035",0.0990379680804718,0.617674928886782,0.610779036538763,"7849/2892/8091/7490",4 |
| hsa00430,"hsa00430","Taurine and hypotaurine metabolism","1/76","11/8035",0.0993201476143791,0.617674928886782,0.610779036538763,"124975",1 |
| hsa00240,"hsa00240","Pyrimidine metabolism","2/76","57/8035",0.101074079272382,0.617674928886782,0.610779036538763,"4907/978",2 |
| hsa05014,"hsa05014","Amyotrophic lateral sclerosis (ALS)","2/76","57/8035",0.101074079272382,0.617674928886782,0.610779036538763,"4744/2904",2 |
| hsa05217,"hsa05217","Basal cell carcinoma","2/76","63/8035",0.119376342097922,0.621239230448363,0.614303545259944,"81029/7476",2 |
| hsa00230,"hsa00230","Purine metabolism","3/76","130/8035",0.124768643446723,0.621239230448363,0.614303545259944,"108/4907/7498",3 |
| hsa04728,"hsa04728","Dopaminergic synapse","3/76","132/8035",0.128981268615109,0.621239230448363,0.614303545259944,"2892/7054/2904",3 |
| hsa00830,"hsa00830","Retinol metabolism","2/76","67/8035",0.13200321280331,0.621239230448363,0.614303545259944,"6121/339761",2 |
| hsa00010,"hsa00010","Glycolysis / Gluconeogenesis","2/76","68/8035",0.135207094686546,0.621239230448363,0.614303545259944,"2645/3948",2 |
| hsa05230,"hsa05230","Central carbon metabolism in cancer","2/76","69/8035",0.138428592881334,0.621239230448363,0.614303545259944,"2645/3948",2 |
| hsa00730,"hsa00730","Thiamine metabolism","1/76","16/8035",0.141184324675197,0.621239230448363,0.614303545259944,"250",1 |
| hsa04917,"hsa04917","Prolactin signaling pathway","2/76","70/8035",0.141667099916704,0.621239230448363,0.614303545259944,"2645/7054",2 |
| hsa04550,"hsa04550","Signaling pathways regulating pluripotency of stem cells","3/76","143/8035",0.153020585335477,0.621239230448363,0.614303545259944,"81029/7044/7476",3 |
| hsa04918,"hsa04918","Thyroid hormone synthesis","2/76","74/8035",0.154779401685957,0.621239230448363,0.614303545259944,"108/7849",2 |
| hsa00511,"hsa00511","Other glycan degradation","1/76","18/8035",0.157387024535374,0.621239230448363,0.614303545259944,"129807",1 |
| hsa04971,"hsa04971","Gastric acid secretion","2/76","76/8035",0.161422507955695,0.621239230448363,0.614303545259944,"108/3758",2 |
| hsa05224,"hsa05224","Breast cancer","3/76","147/8035",0.162097532560669,0.621239230448363,0.614303545259944,"81029/2250/7476",3 |
| hsa04514,"hsa04514","Cell adhesion molecules (CAMs)","3/76","148/8035",0.164392397868829,0.621239230448363,0.614303545259944,"22854/1000/3897",3 |
| hsa04261,"hsa04261","Adrenergic signaling in cardiomyocytes","3/76","149/8035",0.166697168049566,0.621239230448363,0.614303545259944,"108/146/6262",3 |
| hsa05226,"hsa05226","Gastric cancer","3/76","149/8035",0.166697168049566,0.621239230448363,0.614303545259944,"81029/2250/7476",3 |
| hsa04721,"hsa04721","Synaptic vesicle cycle","2/76","78/8035",0.168117700991169,0.621239230448363,0.614303545259944,"23025/6538",2 |
| hsa00983,"hsa00983","Drug metabolism - other enzymes","2/76","79/8035",0.171483500983464,0.621239230448363,0.614303545259944,"7498/978",2 |
| hsa00100,"hsa00100","Steroid biosynthesis","1/76","20/8035",0.173287986098607,0.621239230448363,0.614303545259944,"8435",1 |
| hsa04921,"hsa04921","Oxytocin signaling pathway","3/76","154/8035",0.178363556548299,0.621239230448363,0.614303545259944,"108/3768/6262",3 |
| hsa04934,"hsa04934","Cushing syndrome","3/76","155/8035",0.180724139766796,0.621239230448363,0.614303545259944,"108/81029/7476",3 |
| hsa04310,"hsa04310","Wnt signaling pathway","3/76","160/8035",0.192654210142173,0.636733586311528,0.629624917883807,"81029/7476/1501",3 |
| hsa00515,"hsa00515","Mannose type O-glycan biosynthesis","1/76","23/8035",0.196585772681718,0.636733586311528,0.629624917883807,"146664",1 |
| hsa04260,"hsa04260","Cardiac muscle contraction","2/76","87/8035",0.198780249075549,0.636733586311528,0.629624917883807,"10345/6262",2 |
| hsa04512,"hsa04512","ECM-receptor interaction","2/76","88/8035",0.202231443267581,0.636733586311528,0.629624917883807,"3909/3918",2 |
| hsa04727,"hsa04727","GABAergic synapse","2/76","89/8035",0.205689800523628,0.636733586311528,0.629624917883807,"108/6538",2 |
| hsa05225,"hsa05225","Hepatocellular carcinoma","3/76","168/8035",0.212142370715457,0.636733586311528,0.629624917883807,"8193/81029/7476",3 |
| hsa04970,"hsa04970","Salivary secretion","2/76","91/8035",0.21262626466488,0.636733586311528,0.629624917883807,"108/146",2 |
| hsa05222,"hsa05222","Small cell lung cancer","2/76","92/8035",0.2161035202027,0.636733586311528,0.629624917883807,"3909/3918",2 |
| hsa05414,"hsa05414","Dilated cardiomyopathy (DCM)","2/76","96/8035",0.230063102382412,0.644848455618671,0.637649190563917,"108/6262",2 |
| hsa05231,"hsa05231","Choline metabolism in cancer","2/76","98/8035",0.237067692443934,0.644848455618671,0.637649190563917,"6582/6580",2 |
| hsa04061,"hsa04061","Viral protein interaction with cytokine and cytokine receptor","2/76","100/8035",0.244084759798109,0.644848455618671,0.637649190563917,"11009/8793",2 |
| hsa04360,"hsa04360","Axon guidance","3/76","181/8035",0.244667279062175,0.644848455618671,0.637649190563917,"81029/22854/3897",3 |
| hsa05146,"hsa05146","Amoebiasis","2/76","102/8035",0.251111395081068,0.644848455618671,0.637649190563917,"3909/3918",2 |
| hsa00052,"hsa00052","Galactose metabolism","1/76","31/8035",0.255592530375087,0.644848455618671,0.637649190563917,"2645",1 |
| hsa01523,"hsa01523","Antifolate resistance","1/76","31/8035",0.255592530375087,0.644848455618671,0.637649190563917,"2352",1 |
| hsa05034,"hsa05034","Alcoholism","3/76","187/8035",0.259955715323076,0.644848455618671,0.637649190563917,"7054/2904/8345",3 |
| hsa04928,"hsa04928","Parathyroid hormone synthesis, secretion and action","2/76","106/8035",0.265182240971889,0.644848455618671,0.637649190563917,"108/5741",2 |
| hsa00640,"hsa00640","Propanoate metabolism","1/76","34/8035",0.276599430877238,0.644848455618671,0.637649190563917,"3948",1 |
| hsa05020,"hsa05020","Prion diseases","1/76","35/8035",0.283470877353094,0.644848455618671,0.637649190563917,"3552",1 |
| hsa05145,"hsa05145","Toxoplasmosis","2/76","112/8035",0.286293275491703,0.644848455618671,0.637649190563917,"3909/3918",2 |
| hsa04725,"hsa04725","Cholinergic synapse","2/76","113/8035",0.289808391898843,0.644848455618671,0.637649190563917,"108/3768",2 |
| hsa00250,"hsa00250","Alanine, aspartate and glutamate metabolism","1/76","36/8035",0.29027790401824,0.644848455618671,0.637649190563917,"339983",1 |
| hsa00350,"hsa00350","Tyrosine metabolism","1/76","36/8035",0.29027790401824,0.644848455618671,0.637649190563917,"7054",1 |
| hsa00500,"hsa00500","Starch and sucrose metabolism","1/76","36/8035",0.29027790401824,0.644848455618671,0.637649190563917,"2645",1 |
| hsa00760,"hsa00760","Nicotinate and nicotinamide metabolism","1/76","37/8035",0.297021106830418,0.644848455618671,0.637649190563917,"4907",1 |
| hsa04960,"hsa04960","Aldosterone-regulated sodium reabsorption","1/76","37/8035",0.297021106830418,0.644848455618671,0.637649190563917,"3758",1 |
| hsa05143,"hsa05143","African trypanosomiasis","1/76","37/8035",0.297021106830418,0.644848455618671,0.637649190563917,"8542",1 |
| hsa05216,"hsa05216","Thyroid cancer","1/76","37/8035",0.297021106830418,0.644848455618671,0.637649190563917,"7849",1 |
| hsa00620,"hsa00620","Pyruvate metabolism","1/76","39/8035",0.31031839757845,0.657573496528377,0.650232165626146,"3948",1 |
| hsa04919,"hsa04919","Thyroid hormone signaling pathway","2/76","119/8035",0.310852925631596,0.657573496528377,0.650232165626146,"89869/7068",2 |
| hsa04015,"hsa04015","Rap1 signaling pathway","3/76","210/8035",0.319498823908204,0.666945281780538,0.659499321696864,"108/2250/2904",3 |
| hsa05332,"hsa05332","Graft-versus-host disease","1/76","41/8035",0.32336740934814,0.666945281780538,0.659499321696864,"3552",1 |
| hsa04114,"hsa04114","Oocyte meiosis","2/76","128/8035",0.342179394745578,0.682528648596814,0.67490871153114,"108/89869",2 |
| hsa04930,"hsa04930","Type II diabetes mellitus","1/76","46/8035",0.354933537632757,0.682528648596814,0.67490871153114,"2645",1 |
| hsa04270,"hsa04270","Vascular smooth muscle contraction","2/76","132/8035",0.355971599776192,0.682528648596814,0.67490871153114,"108/146",2 |
| hsa00600,"hsa00600","Sphingolipid metabolism","1/76","47/8035",0.361070106807861,0.682528648596814,0.67490871153114,"129807",1 |
| hsa00520,"hsa00520","Amino sugar and nucleotide sugar metabolism","1/76","48/8035",0.367149059221807,0.682528648596814,0.67490871153114,"2645",1 |
| hsa05322,"hsa05322","Systemic lupus erythematosus","2/76","136/8035",0.369664758179463,0.682528648596814,0.67490871153114,"2904/8345",2 |
| hsa04371,"hsa04371","Apelin signaling pathway","2/76","137/8035",0.37307117191345,0.682528648596814,0.67490871153114,"108/6262",2 |
| hsa00270,"hsa00270","Cysteine and methionine metabolism","1/76","49/8035",0.373170928697097,0.682528648596814,0.67490871153114,"3948",1 |
| hsa00510,"hsa00510","N-Glycan biosynthesis","1/76","50/8035",0.379136244176564,0.682528648596814,0.67490871153114,"146664",1 |
| hsa04979,"hsa04979","Cholesterol metabolism","1/76","50/8035",0.379136244176564,0.682528648596814,0.67490871153114,"8435",1 |
| hsa05144,"hsa05144","Malaria","1/76","50/8035",0.379136244176564,0.682528648596814,0.67490871153114,"1440",1 |
| hsa05165,"hsa05165","Human papillomavirus infection","4/76","330/8035",0.380561428308526,0.682528648596814,0.67490871153114,"3909/3918/81029/7476",4 |
| hsa04913,"hsa04913","Ovarian steroidogenesis","1/76","51/8035",0.385045529767369,0.683145294748559,0.675518473276087,"108",1 |
| hsa05010,"hsa05010","Alzheimer disease","4/76","334/8035",0.389238248072154,0.68323735033942,0.675609501133079,"81029/3552/7476/2904",4 |
